# Indole Diterpenes from Mangrove Sediment-Derived Fungus *Penicillium* sp. UJNMF0740 Protect PC12 Cells against 6-OHDA-Induced Neurotoxicity via Regulating the PI3K/Akt Pathway

**DOI:** 10.3390/md21110593

**Published:** 2023-11-14

**Authors:** Xin-Xin Wang, Ze-Long Chen, Jun-Sheng Zhang, Hai-Shan Liu, Ruo-Ping Ma, Xin-Ping Liu, Ming-Yue Li, Di Ge, Jie Bao, Hua Zhang

**Affiliations:** School of Biological Science and Technology, University of Jinan, 336 West Road of Nan Xinzhuang, Jinan 250022, China; wxinxin202202@163.com (X.-X.W.); 201921200870czl@sina.com (Z.-L.C.); bio_zhangjs@ujn.edu.cn (J.-S.Z.); bio_liuhs@ujn.edu.cn (H.-S.L.); 19511573015@163.com (R.-P.M.); liuxinping255@163.com (X.-P.L.); a1196384910@163.com (M.-Y.L.); bio_ged@ujn.edu.cn (D.G.)

**Keywords:** indole diterpenes, *Penicillium* sp., neuroprotection, PI3K/Akt pathway

## Abstract

In our chemical investigation into *Penicillium* sp. UJNMF0740 derived from mangrove sediment, fourteen indole diterpene analogs, including four new ones, are purified by multiple chromatographic separation methods, with their structures being elucidated by the analyses of NMR, HR-ESIMS, and ECD data. The antibacterial and neuroprotective effects of these isolates were examined, and only compounds **6** and **9** exhibited weak antibacterial activity, while compounds **5**, **8**, and **10** showed protective effects against the injury of PC12 cells induced by 6-hydroxydopamine (6-OHDA). Additionally, compound **5** could suppress the apoptosis and production of reactive oxygen species (ROS) in 6-OHDA-stimulated PC12 cells as well as trigger the phosphorylation of PI3K and Akt. Taken together, our work enriches the structural diversity of indole diterpenes and hints that compounds of this skeleton can repress the 6-OHDA-induced apoptosis of PC12 cells via regulating the PI3K/Akt signaling pathway, which provides evidence for the future utilization of this fascinating class of molecules as potential neuroprotective agents.

## 1. Introduction

**Figure 1:**
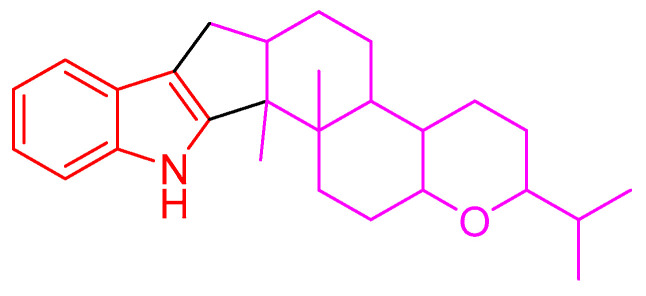
Indole diterpene skeleton.

Indole diterpenes belong to an important class of alkaloids mainly isolated from species of the genera *Penicillium*, *Aspergillus*, *Epichloe*, *Nodulisporium*, and *Tolypocladium*, and they bear a cyclic diterpenoid core originating from geranylgeranyl diphosphate (GGPP) and an indole moiety deriving from tryptophan (Figure 1) [1,2,3]. Indole diterpenes are generally divided into paxilline and non-paxilline types [2,3], and exhibit a range of biological activities involving an inhibition against the big potassium channel receptor [4,5], cholesterol acyl transferase (ACAT) [6], human kinesin [7], nitrogen monoxide (NO) production [8], bacteria [9], cancer cells [10,11], agricultural pests [12], and the H1N1 virus [13], which endows them promising application prospects in the fields of medicine or agriculture. However, the possible detriment to humans or animals has limited their widespread application, as indole diterpenes are often reported to be tremorgenic mycotoxins [1]. Nevertheless, the work on the structural diversity, structure–activity relationship, or biosynthesis of indole diterpenes [1,2,3,14] still attracts considerable attention from the scientific community.

**Figure 2:**
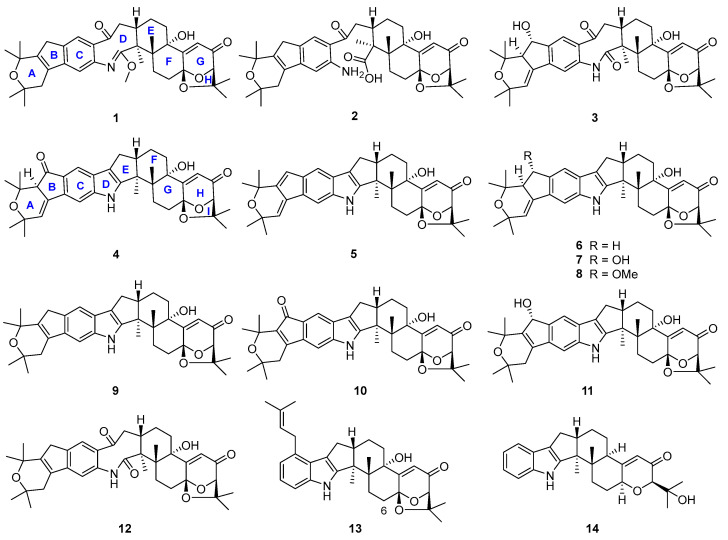
Chemical structures of compounds **1**–**14**.

During our investigation of *Penicillium* sp. UJNMF0740 (the name was revised from *Trichoderma citrinoviride* UJNBHMF0740 [15]) obtained from mangrove sediment, fourteen paxilline-type indole diterpenes, including four new shearinine analogs, were isolated and structurally characterized (Figure 2), and their neuroprotective effects on PC12 cells against injury from 6-hydroxydopamine (6-OHDA), along with antibacterial activities against *Staphylococcus aureus* and *Escherichia coli*, were subsequently evaluated. Among them, compounds **6** and **9** displayed mild growth inhibitory activity against *S. aureus* with IC_50_ values of 48.8 and 47.6 µM, respectively, while compounds **5**, **8**, and **10** significantly alleviated the 6-OHDA-induced injury of PC12 cells. Then, compound **5** was further demonstrated to ameliorate cell damage through inhibiting the production of reactive oxygen species (ROS) and preventing the apoptosis mediated by the PI3K/Akt pathway. In addition, the possible biosynthetic pathway of all the isolated metabolites was proposed. In summary, the current work not only presents new indole diterpene derivatives but also reports the neuroprotective effect of this class of fungal metabolites for the first time, expanding the bioactivity spectrum and providing a new perspective for the future utilization of these interesting alkaloids.

## 2. Results

### 2.1. Structural Elucidation

**Figure 3:**
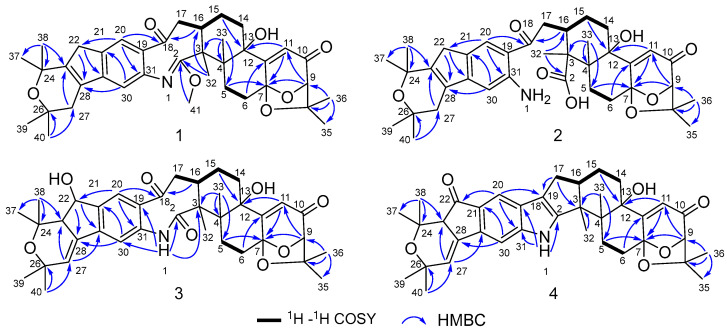
Key ^1^H–^1^H COSY and HMBC correlations of compounds **1**–**4**.

**Figure 4:**
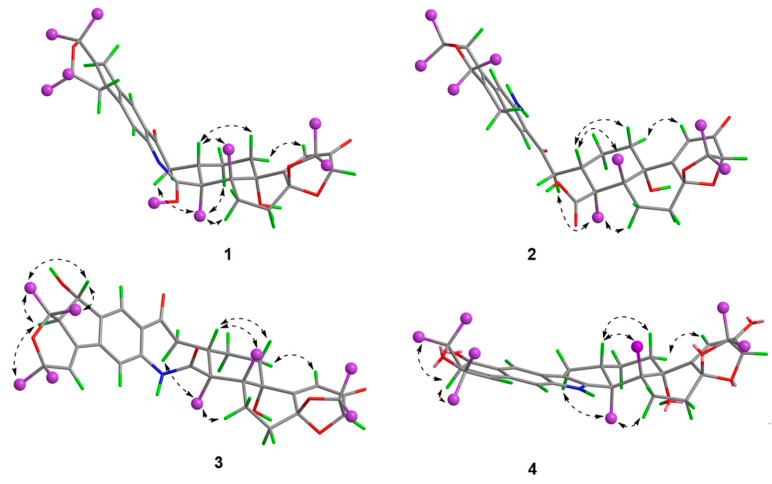
Key NOESY correlations of compounds **1**–**4**.

**Figure 5:**
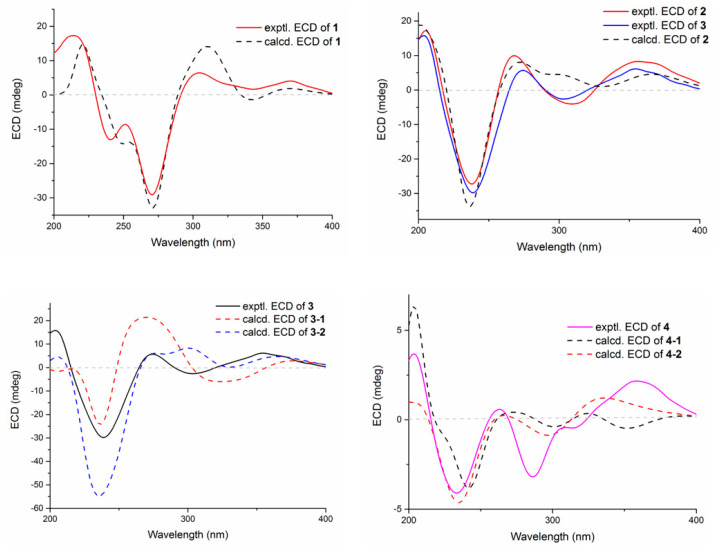
Experimental and calculated ECD spectra of compounds **1**–**4**.

Compound **1** was obtained as an amorphous light-yellow powder, and the molecular formula of C_38_H_47_NO_7_ was deduced from the (+)-HR-ESIMS ion at *m*/*z* 630.3424 ([M + H]^+^, calcd 630.3425) and the 1D NMR data (Table 1), corresponding to 16 degrees of unsaturation. The ^1^H NMR spectrum (Table 1) revealed signals for two aromatic protons (*δ*_H_ 6.55, 7.48), one olefinic proton (*δ*_H_ 5.73), seven methylene groups [*δ*_H_ 1.51, 1.71 (2H), 1.88, 1.95, 2.32, 2.37 (2H), 2.67, 2.75 (2H), 2.91, 3.33 (2H)], two sp^3^ methine protons (*δ*_H_ 3.01, 4.29), eight methyl groups (*δ*_H_ 1.15, 1.16, 1.28, 1.33, 1.41, 1.427, 1.432, 1.61), and one methoxy group (*δ*_H_ 3.64). The ^13^C NMR and DEPT spectra displayed the presence of 38 carbon atoms, assigned as eight methyl carbons (*δ*_C_ 17.6, 23.2, 25.2, 29.0, 29.2, 30.1, 30.2, 30.9), one methoxy carbon (*δ*_C_ 53.3), seven methylene carbons (*δ*_C_ 25.1, 28.2, 28.5, 31.7, 34.5, 36.8, 48.9), five methine carbons (*δ*_C_ 35.2, 88.0, 113.3, 118.2, 123.0), and seventeen quaternary carbons (*δ*_C_ 41.6, 58.2, 71.5, 73.7, 77.3, 78.8, 104.1, 122.3, 131.9, 136.3, 145.0, 149.3, 149.7, 157.9, 169.4, 196.9, 205.3). These 1D (Table 1) and 2D (Figure 3) NMR data greatly resembled those of shearinine H (**12**) [5]. The HMBC correlations from H_3_-36 to C-8/C-9/C-35, from H-9 to C-7/C-10/C-11, from H-11 to C-7/C-13, from H-6 to C-4/C-12, from H-5 to C-7, from H_3_-33 to C-3/C-4/C-5, from H-15 to C-13, from H-16 and H-17 to C-18, and from H_3_-32 to C-2/C-3/C-16, combined with the ^1^H-^1^H COSY correlations of H-16 with H_2_-15 and H_2_-17, and H-14 with H_2_-15, established the diterpenoid moiety on the right, while the HMBC correlations from H-20 to C-18/C-29/C-31, from H-30 to C-19/C-21/C-28, from H-22 to C-21/C-23/C-28/C-29, from H_3_-39 and H_3_-40 to C-26/C-27/C-28, and from H_3_-37 and H_3_-38 to C-23/C-24 suggested the prenylated indole segment on the left (Figure 3), revealing the paxilline-type indole diterpene skeleton of **1** as **12**. However, an additional methoxy group (*δ*_H_/*δ*_C_ 3.64/53.3), the absence of the NH signal (*δ*_H_ 7.20 in **12**), and the remarkably upfield-shifted C-2 signal (Δ*δ*_C_ − 19.2) compared with **12** were observed, indicating that an imine fragment bearing a methoxy group in **1** replaced the amide moiety in **12** [5]. The HMBC correlations from H_3_-32 and H_3_-41 to C-2 further confirmed the location of the methoxy group at C-2. The gross structure of shearinine R (**1**) was therefore constructed as shown in Figure 2. The relative configuration of **1** was deduced by the NOESY spectrum (Figure 4), in which the correlations of H-16/H-14*β*, H-16/H_3_-33, H_3_-32/H-17*α*, H_3_-32/H-5*α*, H_3_-32/H-15*α*, and H-14*α*/H-11 established the fusion of D/E and E/F rings as in **7** [5,16]. The absolute configuration for **1** was determined as shown by comparing the calculated ECD spectrum for the (3*S*,4*R*,7*S*,9*R*,13*S*,16*S*) enantiomer to the experimental one for compound **1** (Figure 5), as well as the biosynthetic considerations [1,2].

Compound **2** was purified as an amorphous light-yellow powder and given a molecular formula of C_37_H_47_NO_8_ based on the protonated molecular ion peak at *m*/*z* 634.3370 ([M + H]^+^, calcd 634.3374) in HR-ESIMS and the 1D NMR data (Table 1), accounting for 15 degrees of unsaturation. The similarity of the ^1^H, ^13^C NMR data for **2** to those for shearinine H (**12**) [5] disclosed that compound **2** was also an indole diterpenoid derivative, while the main differences between **2** and **12** were attributed to the ^13^C chemical shifts of the C ring and C-2/C-3/C-17. A further analysis of the 2D NMR data for **2** (Figure 3) afforded identical structural units of A–C and E–H rings with **12**. The planar structure of **2** was finally elucidated as a D ring opening analog derived from the amide bond hydrolysis of **12**, based on the extra 18 mass units and the loss of one degree of unsaturation compared with **12**. Although direct signals for the free amino and carboxylic acid groups in compound **2** were not observed in the ^1^H NMR spectrum, the ^13^C NMR data for related carbons in **2** were in good agreement with those of shearinine Q [17], an indole diterpenoid analog with the same ring-opening structural fragment, which further corroborated the structure of **2**. Compound **2** shared similar NOESY data (Figure 4) as those of **1**, and the experimental ECD spectrum matched well with the calculated spectrum of the (3*S*,4*R*,7*S*,9*R*,13*S*,16*S*) enantiomer. Therefore, the stereochemistry of compound **2** was deduced as depicted in Figure 2.

The molecular formula C_37_H_45_NO_8_ of compound **3** with 16 degrees of unsaturation was evidenced by the HR-ESIMS ion at *m*/*z* 632.3220 ([M + H]^+^, calcd 632.3218) and the 1D NMR data (Table 1). The NMR signals of **3** highly resembled those reported for shearinine I [5], and the main difference between the two compounds was attributed to the fact that a methylene group (*δ*_H_ 2.63/3.08; *δ*_C_ 33.1) in shearinine I [5] was replaced by an oxygenated methine (*δ*_H_ 4.91; *δ*_C_ 76.3) in **3**, allowing the location of an additional hydroxy group at C-22. The HMBC correlations from H-20 to C-22, and from H-22 to C-21, C-23, C-24, C-28, and C-29 (Figure 3), further confirmed the hydroxylated methine moiety of C-22.

The relative configuration of **3** was elucidated by NOESY correlations (Figure 4). The observed correlations of H-16/H-14*β*, H-16/H_3_-33, H_3_-32/H-17*α*, H_3_-32/H-5*α*, H_3_-32/H-15*α*, and H-14*α*/H-11 were identical to those of compounds **1** and **2**, hinting that **1**–**3** possessed the same relative configurations in the D–H rings. The NOESY cross-peaks of H-22/H_3_-37, H-22/H_3_-38, H-23/H_3_-37, and H-23/H_3_-39 indicated the opposite directions for H-22 and H-23, which was also verified by comparing the NMR data of C-22 and C-23 with those reported for the two *trans* and *cis* isomers (*δ*_H_/*δ*_C_ 76.4/4.96, 60.1/2.67 for *trans-* and 74.3/5.35, 52.5/2.90 for *cis-*) [18]. The absolute configuration for D–H rings of **3** was considered to be consistent with that of **2** on the grounds of their similar ECD curves (Figure 5), owing to the fact that their cotton effects were mainly related to the α,β-unsaturated carbonyl chromophore in the shearinine analogs [17]. Unfortunately, the absolute configurations for C-22 and C-23 could not be determined by the Mosher ester method because of the scanty sample, and they were tentatively deduced as 22*S*,23*R* based on a better ECD match with the calculated one for the (3*S*,4*R*,7*S*,9*R*,13*S*,16*S*,22*S*,23*R*) isomer (**3-1**) than with that for the (3*S*,4*R*,7*S*,9*R*,13*S*,16*S*,22*R*,23S) isomer (**3-2**) (Figure 5). Nonetheless, more sufficient evidence, like the Mosher ester method, can be used to further confirm the absolute configurations for C-22 and C-23.

Compound **4** was revealed to possess a molecular formula of C_37_H_43_NO_6_ according to the HR-ESIMS ion peak at *m*/*z* 598.3166 ([M + H]^+^, calcd 598.3163) and the 1D NMR data (Table 1), corresponding to 17 degrees of unsaturation. The NMR data for **4** were very close to those of shearinine D (**7**) [5], indicating that their structures were highly related. However, a carbonyl signal (*δ*_C_ 201.7) in **4** was observed instead of that for the oxymethine (*δ*_H_ 4.86; *δ*_C_ 76.7) in **7**, indicating that the methine (C-22) in **7** was oxidized to carbonyl in **4**, which was further verified by the HMBC correlations from H-20 and H-23 to C-22, along with the one-less-degree of unsaturation of **4** compared with that of **7**.

The relative configurations of E–I rings for **4** were suggested to be identical with those of **1**–**3** in view of their similar NOESY correlations (Figure 4), while the spatial relationship of H-23 with E–I rings was unable to be defined due to their long distance. Then, the theoretical ECD spectra of the (3*S*,4*R*,7*S*,9*R*,13*S*,16*S*,23*R*) isomer (**4-1**) and (3*S*,4*R*,7*S*,9*R*,13*S*,16*S*,23*S*) isomer (**4-2**) were calculated, and the absolute configuration of **4** was determined as 3*S*,4*R*,7*S*,9*R*,13*S*,16*S*,23*S* based on its consistent experimental ECD curve with that of **4-2**.

Structures of the known compounds **5**–**14** were identified to be 22,23-dehydro-shearinine A (**5**) [19], shearinine A (**6**) [5,20], shearinines D–G (**7**–**10**) [5,18], 22-hydroxylshearinine F (**11**) [16], shearinine H (**12**) [5], paspalitrem C (**13**) [21], and dehydroxypaxilline (**14**) [22] by comparing their NMR data with those reported previously.

**Scheme 1:**
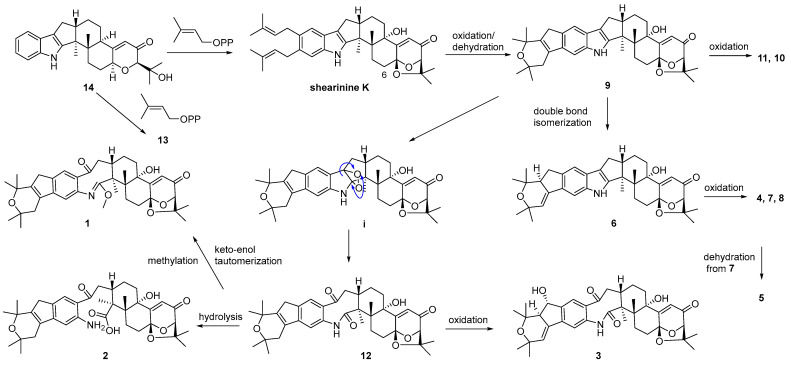
Proposed biosynthetic pathway for compounds **1**–**14**.

A plausible biosynthetic pathway for **1**–**14** was postulated as shown in Scheme 1. Biosynthetically, compounds **13** and shearinine K could be produced through oxidation at C-13, as well as further prenylation and diprenylation on the aromatic ring of **14**, respectively. Subsequently, oxidation and dehydration cyclization on the isopentenyls of shearinine K would afford **9**, while the further oxidation at C-22 of **9** would proceed easily to generate **11** and **10**. The double-bond isomerization from Δ^23,28^ of **9** to Δ^27,28^ would lead to **6**, while the following oxidation and methylation occurring at C-22 of **6** would afford **7**, **4**, and **8**. The oxidation of the respective intermediate (**i**) by the cleavage of the C-2–C-18 bond can produce an eight-membered ring fragment in **12**, while the progress was reported to be automated [5]. Then, the double-bond isomerization, like from **9** to **6**, and the oxidation at C-22 in **12** would form **3**, and **5** would be derived from the dehydration of **7**. Finally, compound **12** could undergo the hydrolysis of the amide bond to yield **2**, and keto-enol tautomerization and the methylation of **12** would produce **1**.

### 2.2. Antimicrobial Activities

The antibacterial activities of compounds **1**–**14** against Gram-positive *Staphylococcus aureus* ATCCC 25923 and Gram-negative *Escherichia coli* ATCC8739 were tested, and only **6** and **9** exhibited weak growth inhibitory effects against *S. aureus* with IC_50_ values of 48.8 and 47.6 µM, respectively, and ceftriaxone sodium was used as a positive control with an IC_50_ < 1.0 µM.

### 2.3. Neuroprotective Effects

**Figure 6:**
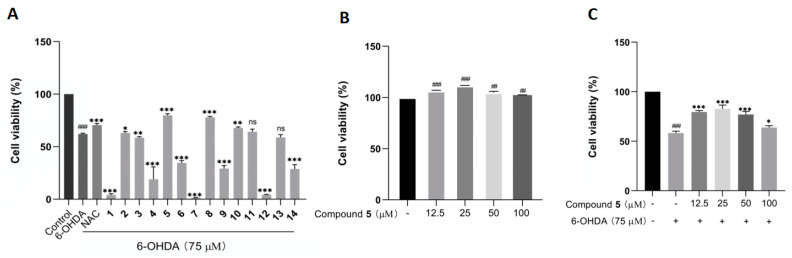
The effects of compounds **1**–**14** on the cell viability of PC12 cells by the MTT assay. (**A**) The cell viability of PC12 cells pretreated with **1**–**14** at 50 µM before exposure to 75 µM 6-OHDA for 12 h. (**B**,**C**) The cell viability of PC12 cells cultured with **5** alone (**B**) or pretreated with different concentrations of **5** before incubation with 6-OHDA (**C**). The data are presented as means ± SD (n = 3), ^##^ *p* < 0.01, and ^###^ *p* < 0.001 compared with the control group; * *p* < 0.05, ** *p* < 0.01, *** *p* < 0.001, and ns (no significance) compared with the 6-OHDA-treated group.

The protective effects against 6-OHDA-induced neuronal injury on PC12 cells of the isolated **1**–**14** were evaluated by an MTT assay with *N*-acetylcysteine (NAC) as reference compound. In the initial test at 50 µM, compounds **5**, **8**, and **10** remarkably rescued the cell viability, with **5** showing the best activity (Figure 6A). In addition, **5** did not exhibit any obvious cytotoxicity toward the PC12 cells within the concentration range from 12.5 to 100 μM, and it even had a certain promoting effect on cell proliferation (Figure 6B). Additionally, compound **5** at 25 μM displayed the best ameliorating effect against the damage of PC12 cells within the testing range (Figure 6C).

This is the first manuscript describing the neuroprotective activity of diterpenoid indole alkaloids. As for the structure–activity relationship (SAR) for compounds **1**–**14**, there was no regular pattern for this group of molecules, as reported for many classes of natural products. Nevertheless, it is interesting to note that several isolates, such as **1**, **7**, and **12**, even displayed significant toxicity to PC12 cells at the testing concentration (50 μM). Particularly, the cases of **7** and **8** demonstrated the surprising effect of methylation in the conversion from toxicity to protection, which further revealed the complexity of the mechanism of natural products.

**Figure 7:**
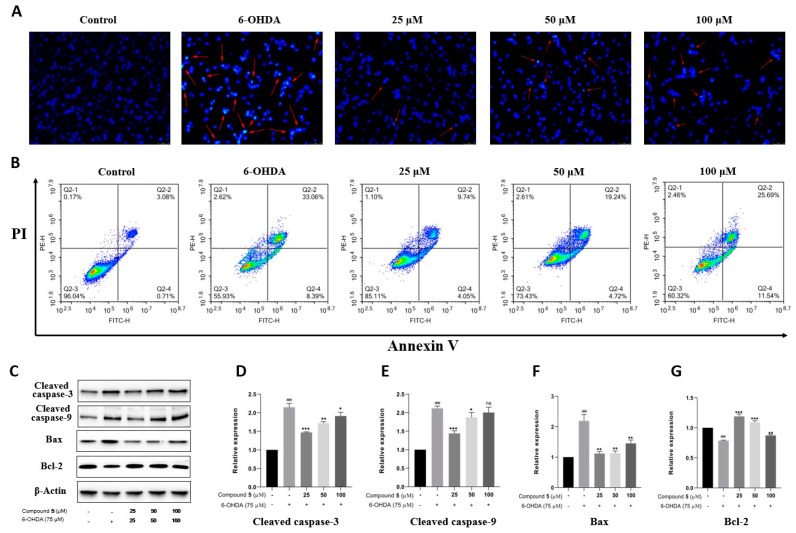
Effect of compound **5** on 6-OHDA-induced cell apoptosis in PC12 cells. Cells were pretreated with different concentrations of **5** and then exposed to 6-OHDA for 12 h. (**A**) Morphology of nuclear chromatin was observed by Hoechst 33258 staining using a fluorescence microscope, while the red arrows mean representative chromatin condensations in nuclei. (**B**) The apoptosis rates were detected by an Annexin V-FITC/PI apoptosis detection kit on flow cytometry. (**C**) The apoptosis-related protein expressions of cleaved caspase-3, cleaved caspase-9, Bax, and Bcl-2 by Western blot. (**D**–**G**) Quantitative analyses of the levels of cleaved caspase-3, cleaved caspase-9, Bax, and Bcl-2 by GraphPad Prism 8.0 software. The values are means ± SDs, ^###^ *p* < 0.001 compared with the control group; * *p* < 0.05, ** *p* < 0.01, *** *p* < 0.001; and ns (no significance) compared with the 6-OHDA-treatment group.

Apoptosis is the main mode of neuronal cell death in many nerve injuries. Hoechst 33258 staining, Annexin V-PI double staining, and Western blot experiments were then performed to assess the effect of **5** on PC12 cell apoptosis induced by 6-OHDA. According to Hoechst 33258 staining, chromatin condensation corresponding to apoptosis was clearly observed in PC12 cells exposed to 6-OHDA, which was notably improved in the groups treated with different concentrations of **5** for 12 h prior to administration with 6-OHDA (Figure 7A). Moreover, the quantitative analysis of cell apoptosis was conducted by double staining with Annexin V-PI on a flow cytometer. As shown in Figure 7B, the proportion of apoptotic cells significantly reduces in the pretreatment groups with compound **5** (13.79%, 23.96%, and 37.23% for 25, 50, and 100 μM, respectively) compared with the control group subjected to 6-OHDA-treatment alone (41.45%).

Several members of the caspase family playing a vital role in apoptosis are often detected as marker proteins, while Bax and Bcl-2 are also important regulatory proteins for mitochondrial permeability in apoptosis [23]. Therefore, the expressions of cleaved caspase-3, cleaved caspase-9, Bax, and Bcl-2 were investigated by Western blotting. As indicated in Figure 7C–G, the exposure of PC12 cells to 6-OHDA leads to the upregulation of cleaved caspase-3, cleaved caspase-9, and Bax, as well as the downregulation of Bcl-2, while pre-treatment with **5** significantly protects PC12 cells against these influences, especially at 25 μM. Collectively, these data provide adequate evidence that compound **5** effectively reduces the apoptosis of PC12 cells stimulated by 6-OHDA.

**Figure 8:**
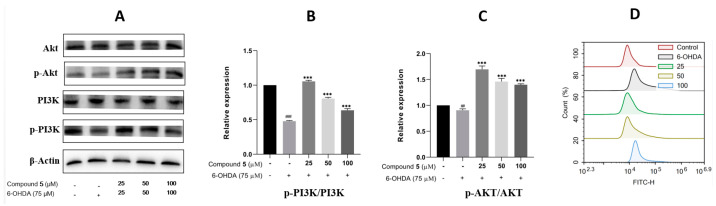
Effects of **5** on the PI3K/Akt pathway and ROS level in PC12 cells. PC12 cells were incubated with different concentrations of **5** for 12 h, and then 6-OHDA (75 μM) was added for an additional 12 h. Protein expressions were detected by Western blot, while the quantification analysis was conducted by GraphPad Prism 8.0 software. (**A**) The protein expressions of Akt, p-Akt, PI3K, and p-PI3K. (**B**,**C**) Quantitative analysis of the ratios of p-PI3K/PI3K and p-Akt/Akt. (**D**) Intracellular ROS was detected by the DCFH-DA reagent and flow cytometry and stated as the fluorescence intensity. All experiments were performed in triplicate. Results are described as means ± SDs, ^##^ *p* < 0.01, ^###^ *p* < 0.001 vs. the control group; *** *p* < 0.001 vs. 6-OHDA-treatment group.

The phosphatidylinositol 3-kinase (PI3K)/protein kinase B (Akt) signaling pathway is well known to play a vital role in multiple cellular processes, such as cell metabolism, proliferation, differentiation, migration, and protein synthesis [24,25]. Thus, we evaluated the effects of 6-OHDA and compound **5** on the expressions of PI3K, p-PI3K, Akt, and p-Akt in PC12 cells. The ratios of p-PI3K/PI3K and p-Akt/Akt were significantly downregulated in the 6-OHDA group compared with the control, while they were upregulated by pre-treatment with **5** (25, 50, and 100 μM) compared with the 6-OHDA group (Figure 8A–C), suggesting that the PI3K/Akt pathway was involved in the suppression of **5** against the PC12 cell injury induced by 6-OHDA.

In neuronal damage, oxidative stress is thought to be an important factor [26]. As 6-OHDA was reported to induce the production of reactive oxygen species (ROS) [27] resulting in cell damage and apoptosis, intracellular ROS was then detected by fluorescence dye DCFH-DA and flow cytometry to examine the effect of **5** on 6-OHDA-induced oxidative damage. The results in Figure 8D indicate that supplementing **5** in 6-OHDA-induced PC12 cells can significantly reduce the intracellular generation of ROS compared with the 6-OHDA group, and the pre-treatment of the 25 μM compound **5** presents the best effect.

## 3. Materials and Methods

### 3.1. General Experimental Procedure

The ESIMS data were measured on an Agilent 6460 Triple Quad LC-MS instrument (Agilent Technologies Inc., Waldbronn, Germany), while the HR-ESIMS spectra were obtained from an Agilent 6545 Q-TOF mass spectrometer (Agilent Technologies Inc., Waldbronn, Germany). The NMR experiments were performed on a Bruker Avance DRX600 spectrometer (Bruker BioSpin AG, Fallanden, Switzerland) with the residual solvent peaks at *δ*_H_ 7.26 and *δ*_C_ 77.2 (CDCl_3_). ECD and UV spectra were acquired on a Chirascan circular dichroism spectrometer with a 1 mm pathway cell (Applied Photophysics Ltd., Surrey, UK). Optical rotations were recorded on a Rudolph VI polarimeter (Rudolph Research Analytical, Hackettstown, USA) with a 10 cm-length cell. Semi-preparative HPLC was performed on a Shimadzu LC-20A instrument (Shimadzu, Tokyo, Japan) with YMC-Pack ODS-A columns (5 μm, 10 × 250 mm, YMC Co. Ltd., Tokyo, Japan). D-101 macroporous resin (Sinopharm Chemical Reagent Co. Ltd., Shanghai, China), silica gel (Qingdao Marine Chemical Co. Ltd., Qingdao, China), Sephadex LH-20 (GE Healthcare Bio-Sciences AB, Uppsala, Sweden), and reversed-phase (RP) C18 (YMC Co. Ltd., Tokyo, Japan) were used for conventional column chromatography (CC). The solvents for CC were of an analytical grade (Tianjin Fuyu Fine Chemical Co. Ltd., Tianjin, China), while the solvents for HPLC were of an HPLC grade (Oceanpak Alexative Chemical Ltd., Goteborg, Sweden).

### 3.2. Fungal Material

The fungus *Penicillium* sp. UJNMF0740 was obtained from the rhizosphere soil of the mangrove plant *Kandelia candel* collected from Dongzhai Harbor (19°95′ N, 110°58′ E), Hainan province. The strain was identified by the sequence of the ITS region with a 99.08% similarity to *Penicillium svalbardense* (NR _111508.1) and deposited at the China General Microbiological Culture Collection Center (CGMCC No.40521).

### 3.3. Fermentation, Extraction, and Isolation

*Penicillium* sp. UJNMF0740 was cultured on potato dextrose broth (PDB) at 28 °C for 3 days. Following this, each of the seed cultures (10 mL) was inoculated into 1 L Erlenmeyer flasks containing rice media. The flasks were then incubated under static conditions at 28 °C for 30 days. After the fermentation process, the rice broth was extracted with 95% ethanol 3 times. The ethanol extract was then evaporated under a reduced pressure to obtain an aqueous solution, which was subsequently extracted with ethyl acetate 5 times to obtain 35 g of crude gum.

The above-described extract was applied to the column chromatography on silica gel with a step gradient of CH_2_Cl_2_-MeOH (*v*/*v* 100:0 → 0:100) to produce 9 fractions (Fr.1–Fr.9). Fr.2 and Fr.3 were chromatographed over a repeated silica gel column using step gradient CH_2_Cl_2_-(CH_3_)_2_CO (*v*/*v* 100:0 → 0:100) to afford seven and ten subfractions, respectively. Fr.2-3 was fractioned by Sephadex LH-20 (CH_2_Cl_2_-MeOH *v*/*v* 1:1) and further purified by semi-prepared HPLC eluting with MeOH-H_2_O (*v*/*v* 55:45, 3.0 mL min^−1^) to yield mixtures of **4** (t_R_ = 12.2 min, 1.5 mg), **8** (t_R_ = 15.0 min, 3.0 mg), and **11** (t_R_ = 15.5 min, 2.2 mg). Fr.3-4 was subjected to Sephadex LH-20 (MeOH) and MPLC on an ODS C18 column to generate eight subfractions (Fr.3-4-1–Fr.3-4-8). Fr.3-4-4 and Fr.3-4-6 were separated by semi-prepared HPLC eluting with MeOH-H_2_O (*v*/*v* 60:40, 3.0 mL min^−1^) to yield **3** (t_R_ = 7.2 min, 1.3 mg) and **7** (t_R_ = 19.1 min, 1.5 mg), and MeOH-H_2_O (*v*/*v* 78:22, 3.0 mL min^−1^) to yield **14** (t_R_ = 11.0 min, 4.5 mg), respectively, while Fr.3-4-8 was purified on a silica gel column and subsequently isolated by semi-prepared HPLC eluting with MeOH-H_2_O (*v*/*v* 81:19, 3.0 mL min^−1^) to yield **9** (t_R_ = 13.8 min, 1.5 mg) and **13** (t_R_ = 13.1 min, 2.3 mg). Moreover, **10** (t_R_ = 13.4 min, 3.5 mg) and **12** (t_R_ = 14.9 min, 4.0 mg) were obtained by semi-prepared HPLC eluting with MeOH-H_2_O (*v*/*v* 91:9, 3.0 mL min^−1^) from Fr.3-4-7. Fr.4 and Fr.6 were separated on a silica gel column using step-gradient CH_2_Cl_2_-MeOH (*v*/*v* 100:0 → 0:100) to afford twelve and eight subfractions, respectively. Fr.4-3 was applied to Sephadex LH-20 (MeOH) and semi-prepared HPLC eluting with MeOH-H_2_O (*v*/*v* 84:16, 3.0 mL min^−1^) to yield **1** (t_R_ = 14.3 min, 1.5 mg), **2** (t_R_ = 17.5 min, 2.3 mg), and **6** (t_R_ = 19.0 min, 4.5 mg), while Fr.6-5 was also purified according to semi-prepared HPLC eluting with MeOH-H_2_O (*v*/*v* 85:15, 3.0 mL min^−1^) to yield **5** (t_R_ = 17.8 min, 1.5 mg).

*Shearinine R* (**1**). Light-yellow powder; [*α*]^25^_D_ 71.8 (*c* 0.69, MeOH); UV (MeOH) *λ*_max_ (log *ε*) 250 (4.14) nm; ECD (0.30 mg mL^–1^, MeOH) *λ* (Δ*ε*) 214 (11.00), 241 (−8.31), 271 (−18.47), 305 (4.08), 370 (2.57) nm; IR *ν*_max_ 3351, 2975, 2937, 1687, 1611, 1435, 1367, 1275, 1196, 1021 cm^–1^; ^1^H and ^13^C NMR data, see Table 1; (+)-ESIMS *m*/*z* 630.2 [M + H]^+^; (+)-HR-ESIMS *m*/*z* 630.3424 [M + H]^+^ (calcd for C_38_H_48_NO_7_, 630.3425).

*Shearinine S* (**2**). Light-yellow powder; [*α*]^25^_D_ 50.8 (*c* 0.26, MeOH); UV (MeOH) *λ*_max_ (log *ε*) 252 (4.26), 303 (4.03) nm; ECD (0.31 mg mL^–1^, MeOH) *λ* (Δ*ε*) 205 (10.59), 238 (−16.76), 268 (6.12), 310 (−2.48), 357 (5.09) nm; ^1^H and ^13^C NMR data, see Table 1; (+)-ESIMS *m*/*z* 634.2 [M + H]^+^; (+)-HR-ESIMS *m*/*z* 634.3370 [M + H]^+^ (calcd for C_37_H_48_NO_8_, 634.3374).

*22-hydroxyshearinine I* (**3**). White powder; [*α*]^25^_D_ 32.4 (*c* 0.13, MeOH); UV (MeOH) *λ*_max_ (log *ε*) 238 (4.25), 294 (3.75) nm; ECD (0.23 mg mL^–1^, MeOH) *λ* (Δ*ε*) 204 (13.14), 239 (−24.78), 274 (4.73), 303 (−2.17), 354 (5.10) nm; IR *ν*_max_ 3420, 2973, 2935, 1686, 1611, 1456, 1385, 1274, 1220, 1026 cm^–1^; ^1^H and ^13^C NMR data, see Table 1; (+)-ESIMS *m*/*z* 632.3 [M + H]^+^; (+)-HR-ESIMS *m*/*z* 632.3220 [M + H]^+^ (calcd for C_37_H_46_NO_8_, 632.3218).

*Shearinine T* (**4**). White powder; [*α*]^25^_D_ 15.7 (*c* 0.23, MeOH); UV (MeOH) *λ*_max_ (log *ε*) 285 (4.31) nm; ECD (0.10 mg mL^–1^, MeOH) *λ* (Δ*ε*) 204 (6.66), 233 (−7.42), 263 (1.04), 286 (−5.77), 358 (3.92) nm; ^1^H and ^13^C NMR data, see Table 1. (+)-ESIMS *m*/*z* 598.3 [M + H]^+^; (+)-HR-ESIMS *m*/*z* 598.3166 [M + H]^+^ (calcd for C_37_H_44_NO_6_, 598.3163).

### 3.4. Assay of Antimicrobial Activity

Liquid growth inhibition in the 96-well microplate assay [28] was applied to evaluate the initially antimicrobial activity against *Staphylococcus aureus* ATCCC 25923 and *Escherichia coli* ATCC8739 strains for the obtained fourteen metabolites at 50 μM, while a 2-fold serial dilution method in 96-well microplates [28] was used to determine the IC_50_ values for the compounds with inhibition rates higher than 50% in the primary test.

### 3.5. Cell Line and Culture

A rat PC12 cell line was obtained from the Procell Life Science & Technology Co., Ltd. (Wuhan, China). The cells were maintained in RPMI-1640 media supplemented with 10% FBS and 1% penicillin/streptomycin (Genview, China) in a humidified incubator with a 5% CO_2_ atmosphere at 37 °C.

### 3.6. Cell Viability Assay

The MTT assay was performed to measure the viability of the PC12 cells with different treatments. PC12 cells were seeded in a 96-well plate at a density of roughly 1.5 × 10^5^ cells per well and cultured for 24 h. Then, the cells were pre-treated with DMSO or the tested compounds for 12 h, and then exposed to 75 μM of 6-OHDA for 12 h. After incubation, the MTT solution was added to the cells with a final concentration of 0.5 mg/mL and further incubated at 37 °C for 4 h. The supernatants were carefully removed and 100 µL of DMSO was added to each well to dissolve formazan crystals. Finally, the absorbance was detected at 490 nm with a Microplate Reader (Tecan, Switzerland). The cell viability was presented as the percentage of the absorbance measured in the vehicle-treated cells.

### 3.7. Hoechst 33258-Staining Assay

Hoechst 33258 staining was used to assess the chromatin condensation, a typical feature of cellular apoptosis. PC12 cells were plated in 6-well plates and treated with different concentrations of compound **5** (25, 50, and 100 μM) for 12 h, and exposed to 75 μM of 6-OHDA for a further 12 h. Then, a 4% paraformaldehyde solution was applied to fix the cells, which were stained with 10 μg/mL of Hoechst 33258 (Sigma-Aldrich, St Louis, MO, USA) for 15 min at 37 °C, followed by washing twice with 1× PBS. The stained cells were observed under a fluorescence microscope (Leica DMI8, Wetzlar, Germany).

### 3.8. Flow Cytometric Analysis

The effect on the apoptosis of PC12 incubated with 6-OHDA from compound **5** was evaluated by Annexin V-FITC/PI double staining associated with flow cytometry. The PC12 cells were first pretreated with different concentrations of **5** and 75 μM of 6-OHDA, as previously indicated. The harvested cells were washed with PBS and resuspended in the binding buffer to be about 1 × 10^6^ cells/mL. Then, the cells were incubated with Annexin V-FITC and PI in the dark for 15 min and detected with a flow cytometer (ACEA Biosciences, San Diego, CA, USA).

### 3.9. Western Blot Assay

The PC12 cells from different treatment groups were washed twice with PBS and collected and lysed with the RIPA lysis buffer (Seven Biotech, Beijing, China). The protein concentrations were assessed with a BCA protein assay kit (Beyotime Biotechnology, Shanghai, China) and equal amounts of protein were separated by 10% SDS-polyacrylamide gel electrophoresis followed by being transferred onto polyvinylidene fluoride membranes (Millipore, Bedford, MA, USA). The membrane was blocked with 5% skimmed milk and incubated overnight at 4 °C with the appropriate primary antibodies: β-actin (1:50,000; ABclonal Technology, Wuhan, China); cleaved caspase-3 (1:1000, Cell Signaling Technology, Danvers, MA, USA); cleaved caspase-9 (1:1000, Cell Signaling Technology); Bax (1:1000, Cell Signaling Technology); Bcl-2 (1:1000, Affinity Biosciences); Akt (1:1000, Cell Signaling Technology); p-Akt (1:1000, Cell Signaling Technology); PI3K (1:1000, Cell Signaling Technology); and p-PI3K (1:1000, Affinity Biosciences). After 3 rinses with Tris-buffered saline containing Tween 20 (TBST), the membranes were incubated with a horseradish peroxidase (HRP)-conjugated secondary antibody (1:5000, ABclonal Technology) for 1 h at room temperature, followed by washing 3 times with TBST again. Finally, the protein bands were imaged using a western enhanced chemiluminescence (ECL) substrate (Merck Millipore, Darmstadt, Germany) and Chemi-Doc XRS system (Bio-Rad, Hercules, CA, USA).

### 3.10. Intracellular ROS Assay

The intracellular ROS level was measured by fluorescence with a 2′,7′-dichloroflurescein diacetate (DCFH-DA) assay. DCFH-DA without fluorescence can pass through the cell membrane and hydrolyze to generate DCFH. DCFH can be oxidized by intracellular ROS to form fluorescent DCF, while the intensity of green fluorescence is proportional to the level of ROS. PC12 cells were cultured in 6-well plates and pre-treated as mentioned above. Then, the cells were washed with PBS and incubated with DCFH-DA at 37 °C for 20 min. ROS generation was measured by the fluorescence derived from the DCF using a flow cytometer (ACEA Biosciences, San Diego, CA, USA) with excitation and emission wavelengths of 488 and 525 nm, respectively.

### 3.11. Statistical Analysis

All the results are expressed as means ± SDs according to at least three independent experiments. One-way ANOVA followed by Tukey’s multiple comparison post hoc test and GraphPad Prism 8.0 software (GraphPad Software Inc., San Diego, CA, USA) were used to analyze the statistical significance. Values of *p* < 0.05 were considered to show a significant difference.

## Figures and Tables

**Table 1:** ^1^H (600 MHz) and ^13^C (150 MHz) NMR data of compounds **1**–**4** in CDCl_3_ (*δ* in ppm).

No.	1		2		3		4	
	*δ*_C_	*δ*_H_ (*J* in Hz)	*δ*_C_	*δ*_H_ (*J* in Hz)	*δ*_C_	*δ*_H_ (*J* in Hz)	*δ*_C_	*δ*_H_ (*J* in Hz)
**1**								7.95, s
**2**	157.9, C		180.0, C		176.8, C		155.4, C	
**3**	58.2, C		53.9, C		57.5, C		52.1, C	
**4**	41.6, C		40.3, C		41.5, C		40.0, C	
**5**	28.2, CH_2_	2.75, m	28.0, CH_2_	2.71, m 2.72, m	27.5, CH_2_	2.85, dd, 15.1, 9.7 1.55, m	27.2, CH_2_	2.70, dd, 12.4, 10.2 1.84, dd, 12.4, 9.7
**6**	28.5, CH_2_	2.67, m 1.95, dd, 12.8, 5.0	28.6, CH_2_	1.59, m 1.91, m	28.5, CH_2_	2.69, dd, 10.4, 7.1 1.95, m	28.4, CH_2_	2.84, m 2.06, m
**7**	104.1, C		104.0, C		104.1, C		104.4, C	
**9**	88.0, CH	4.29, d, 1.2	88.1 CH	4.30, s	88.0, CH	4.28, d, 1.2	88.1, CH	4.33, s
**10**	196.9, C		197.1, C		196.9, C		197.1, C	
**11**	118.2, CH	5.73, brs	118.5, CH	5.84, s	118.4, CH	5.73, s	118.0, CH	5.85, brs
**12**	169.4, C		169.5, C		169.4, C		169.4, C	
**13**	77.3, C		77.4, C		77.4, C		77.8, C	
**14**	31.7, CH_2_	1.71, m	30.9, CH_2_	1.74, m 1.87, m	31.8, CH_2_	1.95, m 1.57, m	34.0, CH_2_	1.99, m 1.92, m
**15**	25.1, CH_2_	1.88, ddd, 26.1, 12.9, 7.8 1.51, m	22.9, CH_2_	1.68, m1.74, m	25.7, CH_2_	1.75, m 1.57, m	21.1, CH_2_	2.09, m 1.83, m
**16**	35.2, CH	3.01, m	33.9, CH	3.07, m	34.6, CH	3.00, m	48.7, CH	2.84, m
**17**	48.9, CH_2_	2.91, dd, 18.6, 6.2 2.32, dd, 18.6, 2.6	43.9, CH_2_	3.02, d, 14.4 2.64, dd, 14.9, 10.4	48.4, CH_2_	3.10, dd, 18.0, 5.3 2.47, dd, 18.0, 3.3	27.6, CH_2_	2.76, dd, 13.2, 6.4 2.47, dd, 13.2, 10.6
**18**	205.3, C		201.2, C		203.0, C		119.2, C	
**19**	122.3, C		115.1, C		135.2, C		127.1, C	
**20**	123.0, CH	7.48, s	125.6, CH	7.91, s	125.5, CH	7.70, s	102.8, CH	7.86, s
**21**	145.0, C		129.9, C		145.2, C		130.5, C	
**22**	36.8, CH_2_	3.33, m	36.4, CH_2_	3.32, s	76.3, CH	4.91, d, 6.3	201.7, C	
**23**	149.3, C		151.8, C		59.8, CH	2.66, dd, 6.3, 3.0	56.0, CH	3.03, d, 2.3
**24**	73.7, C		73.7, C		73.8, C		74.2, C	
**26**	71.5, C		71.5, C		72.6, C		72.8, C	
**27**	34.5, CH_2_	2.37, m	34.3, CH_2_	2.34, brs	127.3, CH	6.15, d, 3.0	124.2, CH	6.20, d, 2.3
**28**	131.9, C		131.9, C		133.0, C		141.1, C	
**29**	136.3, C		151.6, C		142.7, C		131.5, C	
**30**	113.3, CH	6.55, s	106.4, CH	6.47, s	118.7, CH	7.06, s	114.3, CH	7.52, s
**31**	149.7, C		151.1, C		137.7, C		145.2, C	
**32**	17.6, CH_3_	1.61, s	15.5, CH_3_	1.56, s	17.7, CH_3_	1.66, s	16.4, CH_3_	1.42, s
**33**	25.2, CH_3_	1.16, s	24.8, CH_3_	1.23, s	24.6, CH_3_	1.19, s	23.9, CH_3_	1.23, s
**34**	78.8, C		78.7, C		78.8, C		79.0, C	
**35**	23.2, CH_3_	1.15, s	23.2, CH_3_	1.16, s	23.2, CH_3_	1.15, s	23.3, CH_3_	1.19, s
**36**	29.0, CH_3_	1.432, s	30.0, CH_3_	1.41, s	29.0, CH_3_	1.42, s	29.0, CH_3_	1.45, s
**37**	30.9, CH_3_	1.41, s	29.6, CH_3_	1.42, s	23.5, CH_3_	1.15, s	29.8, CH_3_	1.67, s
**38**	30.1, CH_3_	1.427, s	29.6, CH_3_	1.41, s	30.2, CH_3_	1.47, s	22.3, CH_3_	1.00, s
**39**	29.2, CH_3_	1.28, s	30.3, CH_3_	1.30, s	31.6, CH_3_	1.32, s	31.9, CH_3_	1.40. s
**40**	30.2, CH_3_	1.33, s	30.3, CH_3_	1.30, s	29.5, CH_3_	1.36, s	29.9, CH_3_	1.38, s
**41**	53.3, CH_3_	3.64, s						

## Data Availability

The data are presented within the article and Supplementary Materials.

## Supplementary Materials

The following supporting information can be downloaded at: https://www.mdpi.com/article/10.3390/md21110593/s1, Figures S1–S28: 1D/2D NMR and HR-ESIMS spectra of compounds **1**–**4**.
