# Supplementary material for: Indole Diterpenes from Mangrove Sediment-Derived Fungus Penicillium sp. UJNMF0740 Protect PC12 Cells against 6-OHDA-Induced Neurotoxicity via Regulating the PI3K/Akt Pathway
Source: Mar Drugs. 2023 Nov 14;21(11):593. doi: 10.3390/md21110593 (PMC10672321; doi:10.3390/md21110593)

Supporting information **for**

Indole-diterpenes from Mangrove Sediment-Derived Fungus *Penicillium* sp. UJNMF0740 Protect PC12 Cells against 6-OHDA-Induced Neurotoxicity via Regulating the PI3K/Akt Pathway

Xin-Xin Wang†, Ze-Long Chen†, Jun-Sheng Zhang, Haishan Liu, Ruo-Ping Ma, Xin-Ping Liu, Ming-Yue Li, Di Ge, Jie Bao*, Hua Zhang*

School of Biological Science and Technology, University of Jinan, 336 West Road of Nan Xinzhuang, Jinan 250022, China

* Correspondence: bio_baoj@ujn.edu.cn (J.B.); bio_zhangh@ujn.edu.cn (H.Z.);
Tel.: 86-0531-89736199

† These authors contributed equally to this work.

Table of contents

**Figure S1.** The ^{1}H-NMR spectrum of compound **1** in $CDCl_3$	**P1**
**Figure S2.** The ^{13}C-NMR spectrum of compound **1** in $CDCl_3$	**P1**
**Figure S3.** The HSQC spectrum of compound **1** in $CDCl_3$	**P2**
**Figure S4.** The ^{1}H-^{1}H COSY spectrum of compound **1** in $CDCl_3$	**P2**
**Figure S5.** The HMBC spectrum of compound **1** in $CDCl_3$	**P3**
**Figure S6.** The NOESY spectrum of compound **1** in $CDCl_3$	**P3**
**Figure S7.** The (+)-HR-ESIMS spectrum of compound **1**	**P4**
**Figure S8.** The ^{1}H-NMR spectrum of compound **2** in $CDCl_3$	**P4**
**Figure S9.** The ^{13}C-NMR spectrum of compound **2** in $CDCl_3$	**P5**
**Figure S10.** The HSQC spectrum of compound **2** in $CDCl_3$	**P5**
**Figure S11.** The ^{1}H-^{1}H COSY spectrum of compound **2** in $CDCl_3$	**P6**
**Figure S12.** The HMBC spectrum of compound **2** in $CDCl_3$	**P6**
**Figure S13.** The NOESY spectrum of compound **2** in $CDCl_3$	**P7**
**Figure S14.** The (+)-HR-ESIMS spectrum of compound **2**	**P7**
**Figure S15.** The ^{1}H-NMR spectrum of compound **3** in $CDCl_3$	**P8**
**Figure S16.** The ^{13}C-NMR spectrum of compound **3** in $CDCl_3$	**P8**
**Figure S17.** The HSQC spectrum of compound **3** in $CDCl_3$	**P9**
**Figure S18.** The ^{1}H-^{1}H COSY spectrum of compound **3** in $CDCl_3$	**P9**
**Figure S19.** The HMBC spectrum of compound **3** in $CDCl_3$	**P10**
**Figure S20.** The NOESY spectrum of compound **3** in $CDCl_3$	**P10**
**Figure S21.** The (+)-HR-ESIMS spectrum of compound **3**	**P11**
**Figure S22.** The ^{1}H-NMR spectrum of compound **4** in $CDCl_3$	**P11**
**Figure S23.** The ^{13}C-NMR spectrum of compound **4** in $CDCl_3$	**P12**
**Figure S24.** The HSQC spectrum of compound **4** in $CDCl_3$	**P12**
**Figure S25.** The ^{1}H-^{1}H COSY spectrum of compound **4** in $CDCl_3$	**P13**
**Figure S26.** The HMBC spectrum of compound **4** in $CDCl_3$	**P13**
**Figure S27.** The NOESY spectrum of compound **4** in $CDCl_3$	**P14**
**Figure S28.** The (+)-HR-ESIMS spectrum of compound **4**	**P14**

Figure S1. The ^{1}H-NMR spectrum of compound **1** in $CDCl_3$

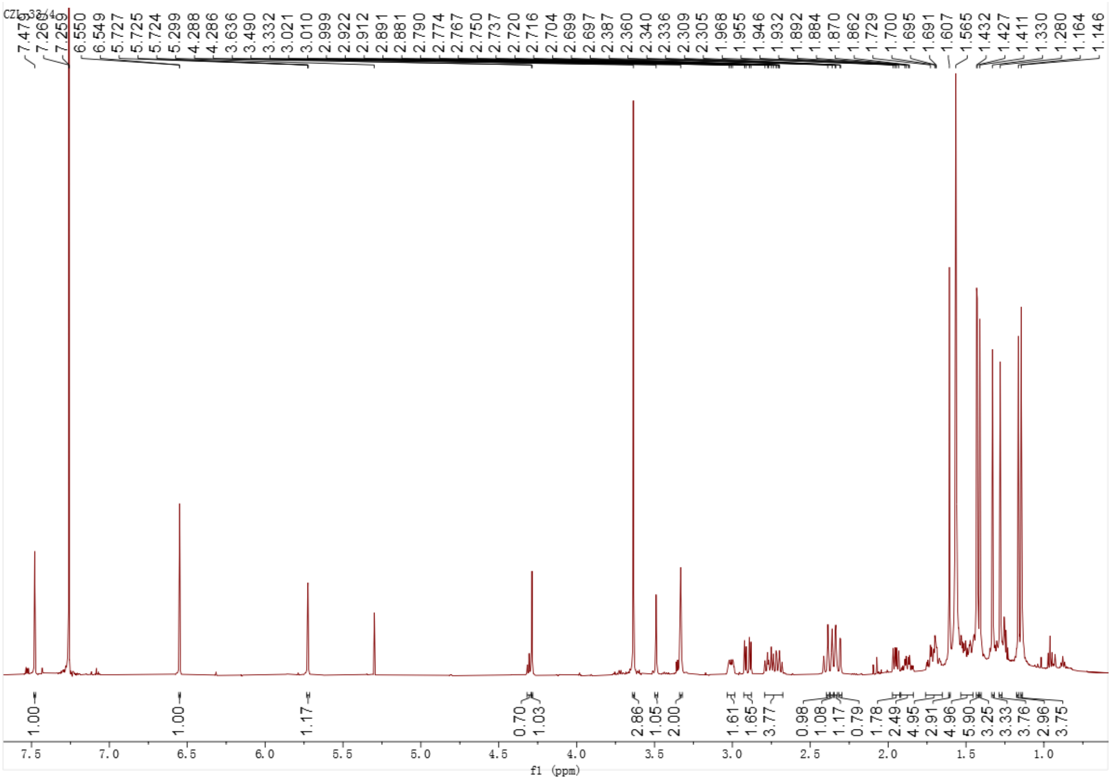

Figure S2. The ^{13}C-NMR spectrum of compound **1** in $CDCl_3$

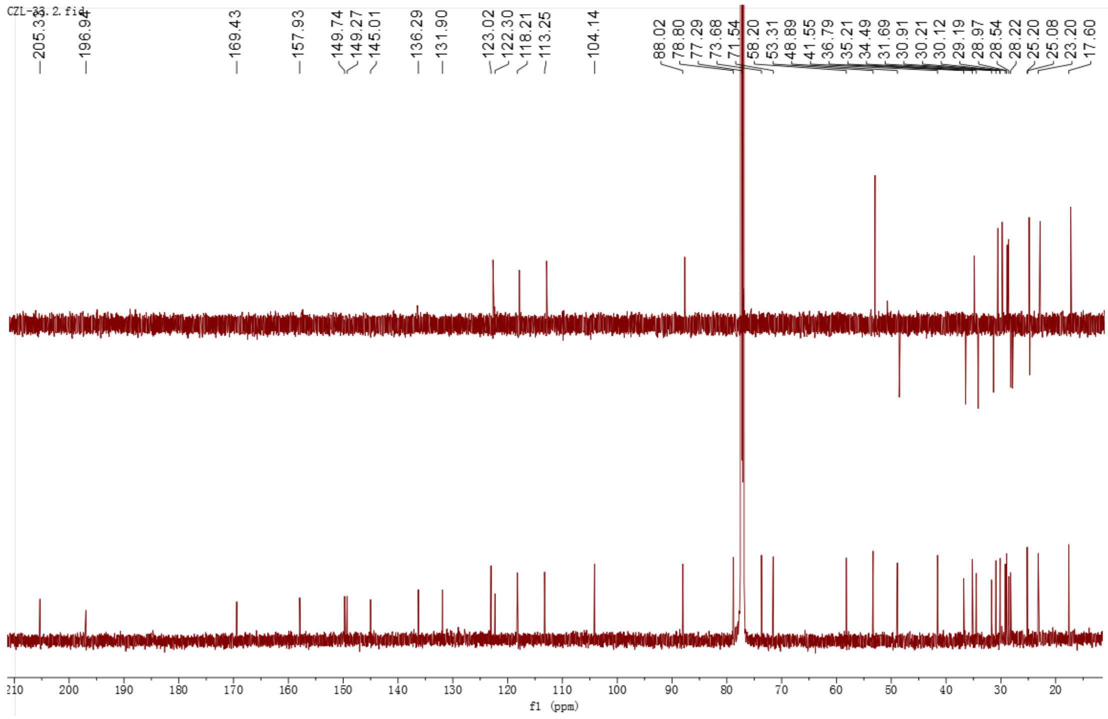

Figure S3. The HSQC spectrum of compound **1** in $CDCl_3$

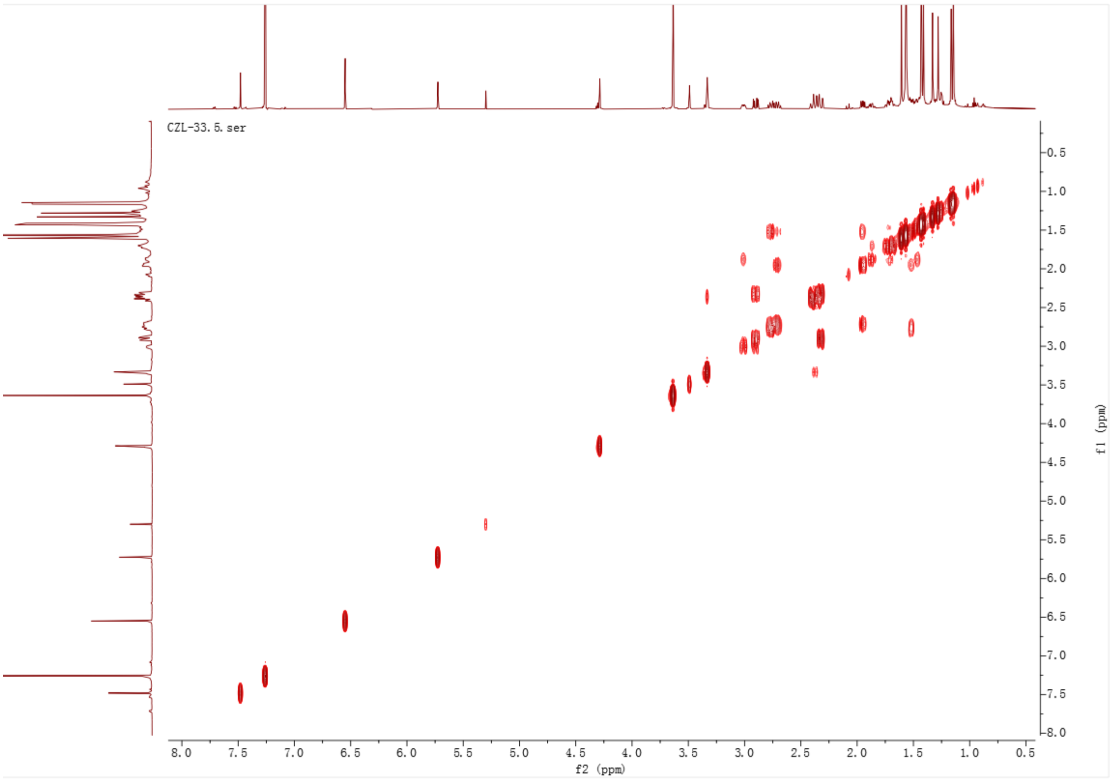

Figure S4. The ^{1}H-^{1}H COSY spectrum of compound **1** in $CDCl_3$

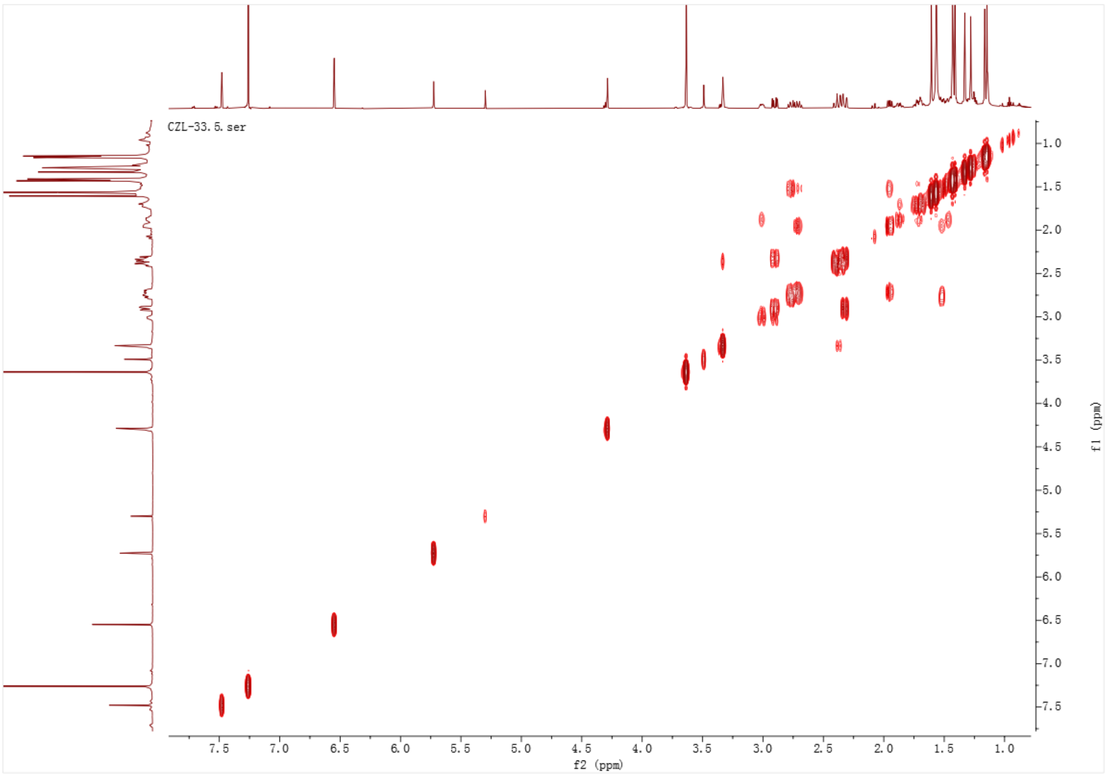

Figure S5. The HMBC spectrum of compound **1** in $CDCl_3$

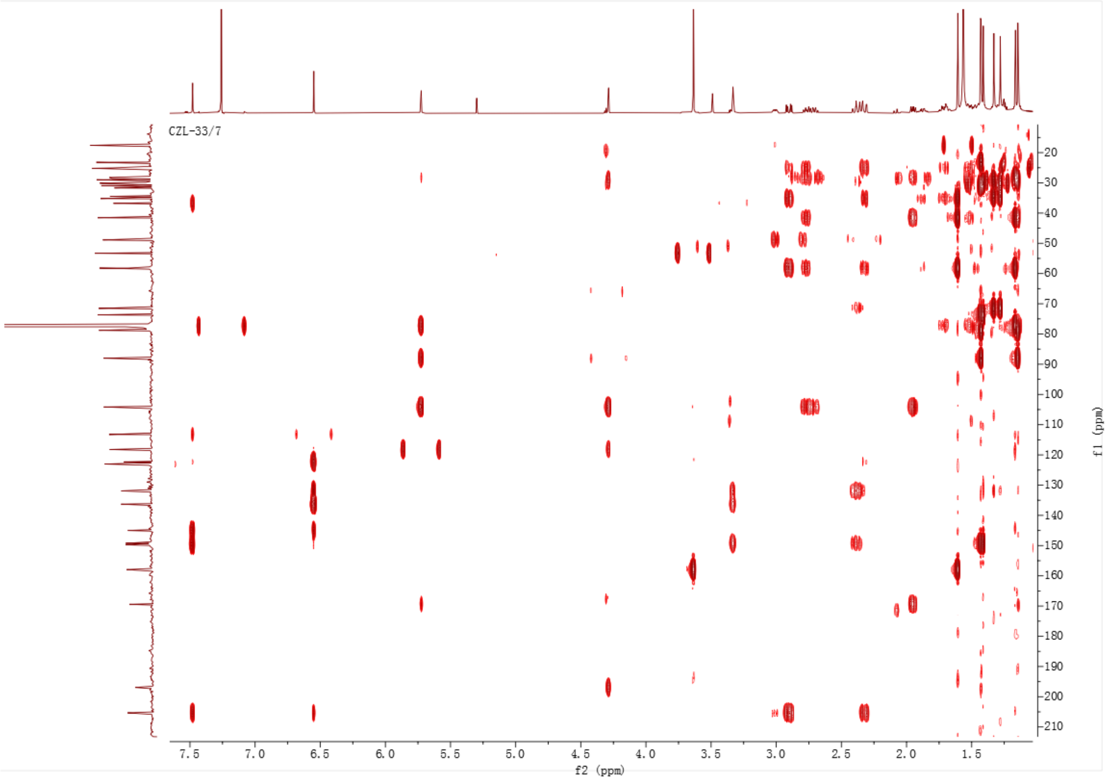

Figure S6. The NOESY spectrum of compound **1** in $CDCl_3$

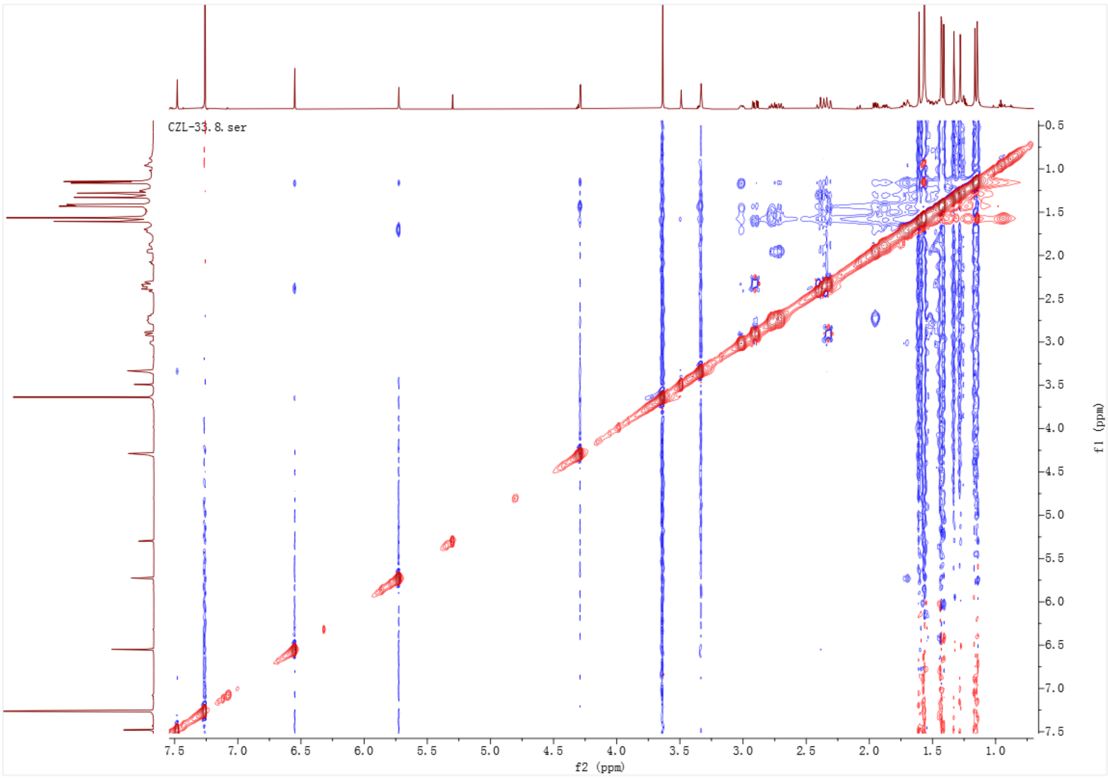

Figure S7. The (+)-HR-ESIMS spectrum of compound **1**

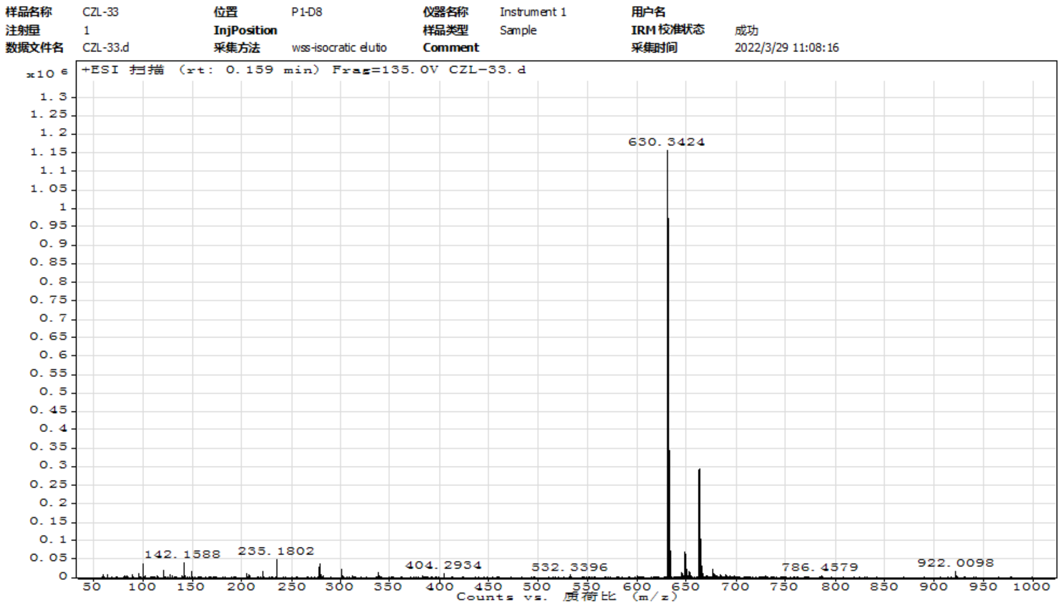

Figure S8. The ^{1}H-NMR spectrum of compound **2** in $CDCl_3$

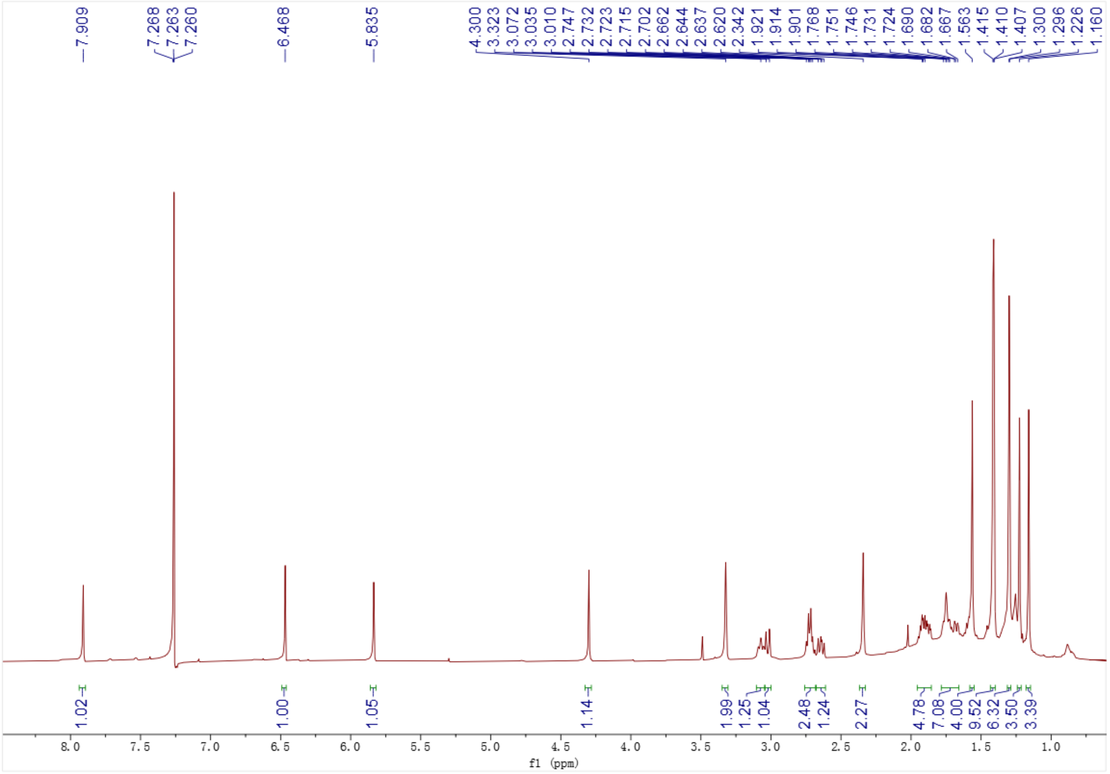

Figure S9. The ^{13}C-NMR spectrum of compound **2** in $CDCl_3$

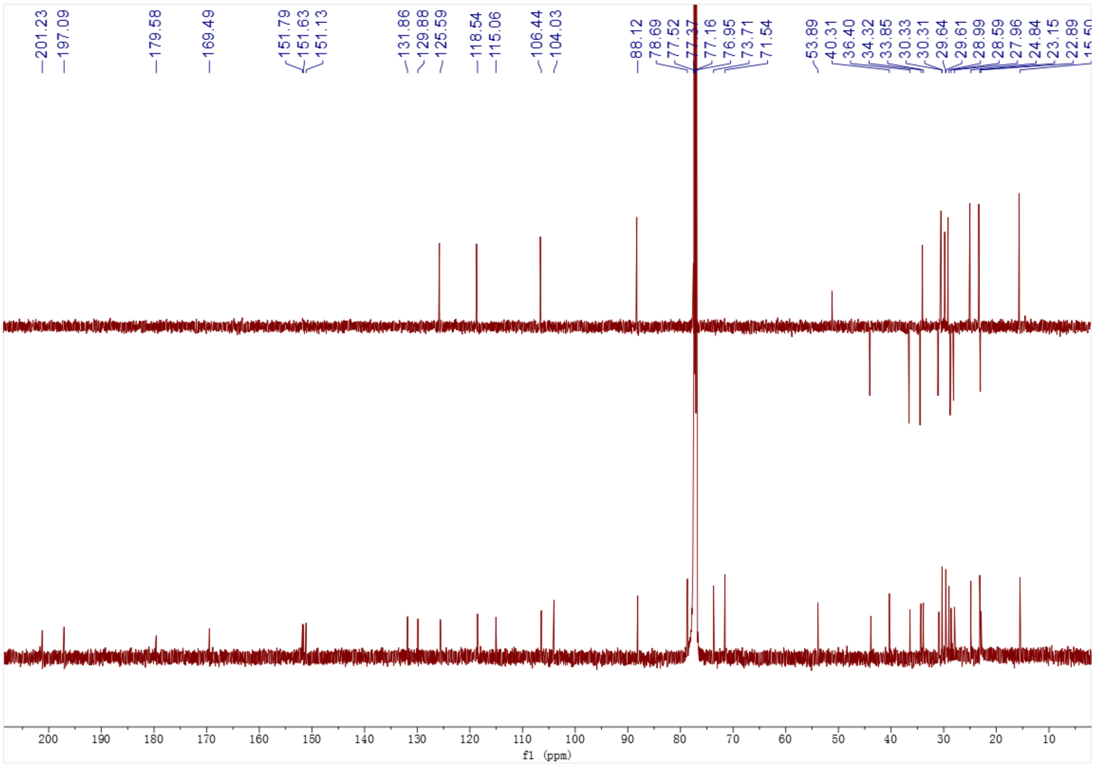

Figure S10. The HSQC spectrum of compound **2** in $CDCl_3$

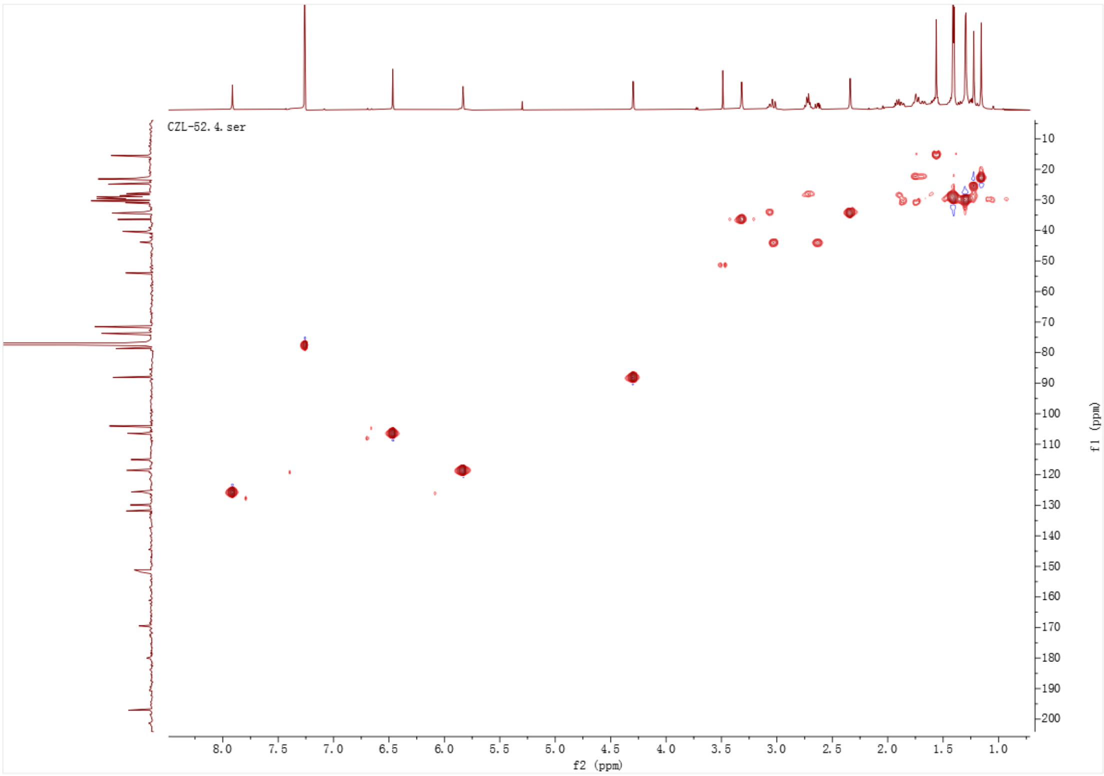

Figure S11. The ^{1}H-^{1}H COSY spectrum of compound **2** in $CDCl_3$

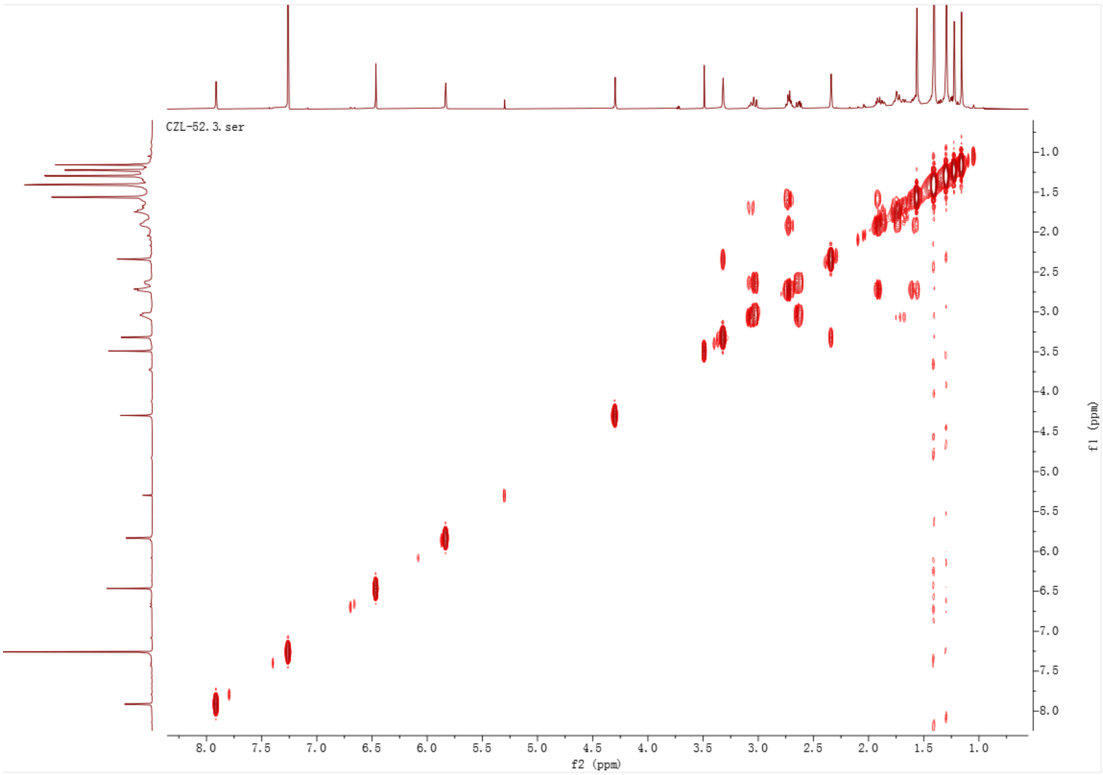

Figure S12. The HMBC spectrum of compound **2** in $CDCl_3$

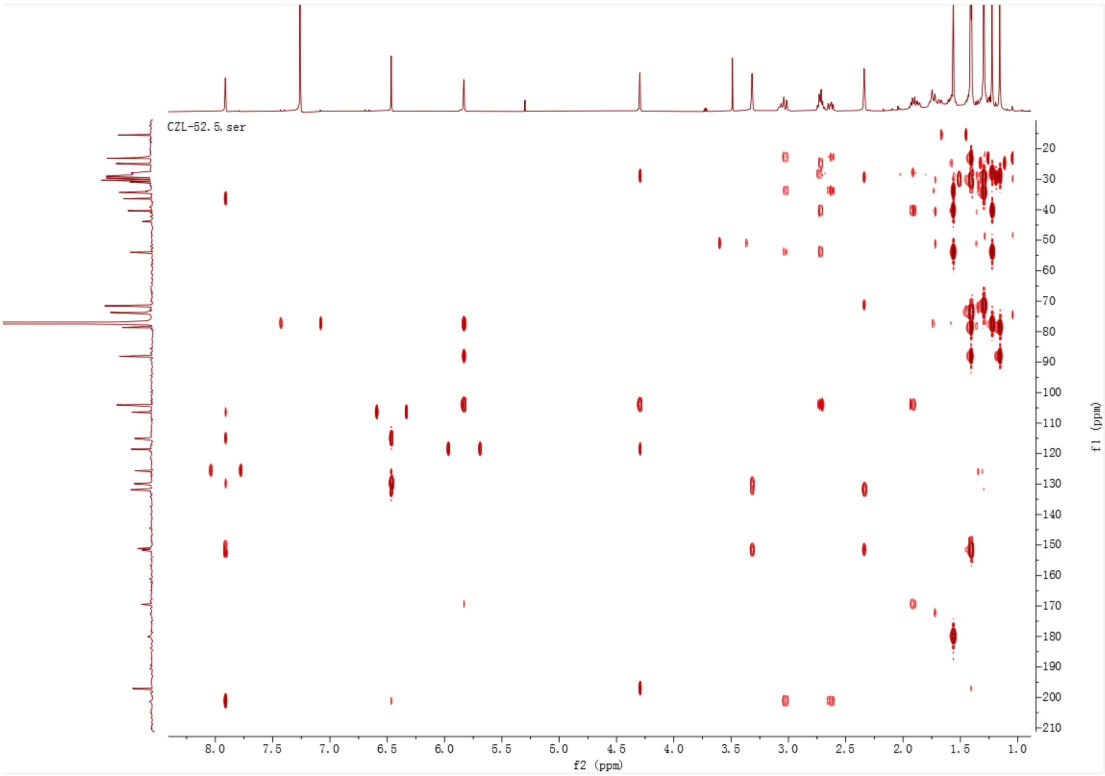

Figure S13. The NOESY spectrum of compound **2** in $CDCl_3$

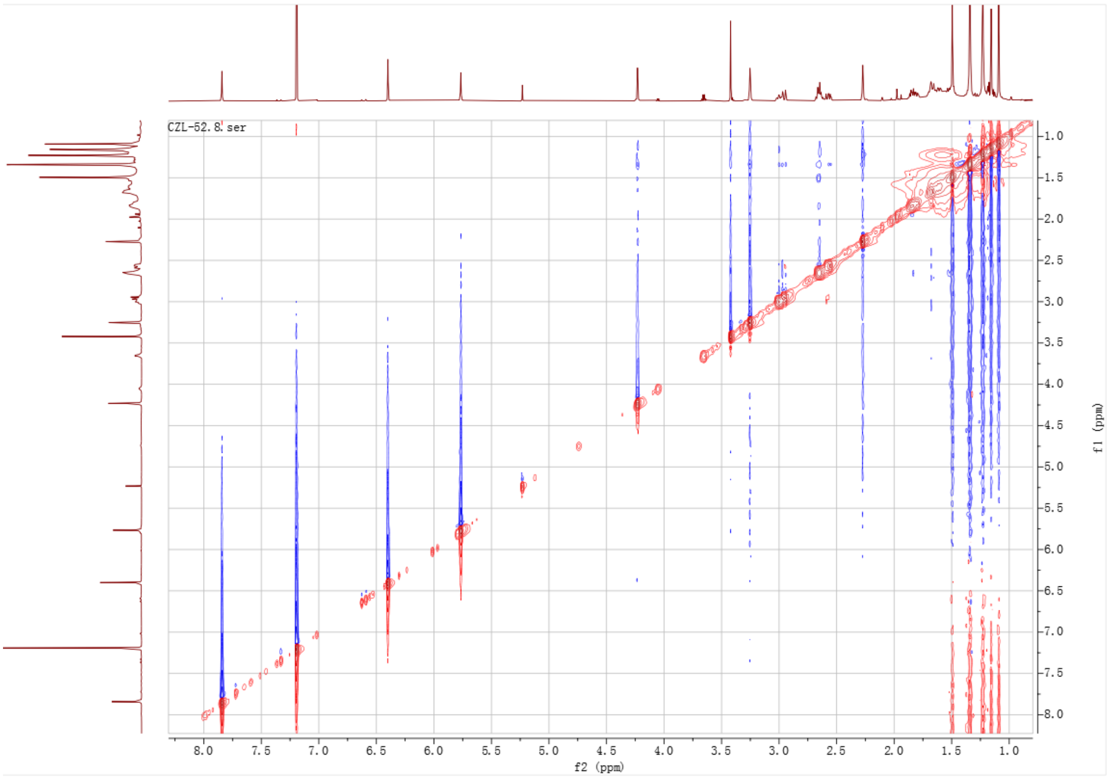

Figure S14. The (+)-HR-ESIMS spectrum of compound **2**

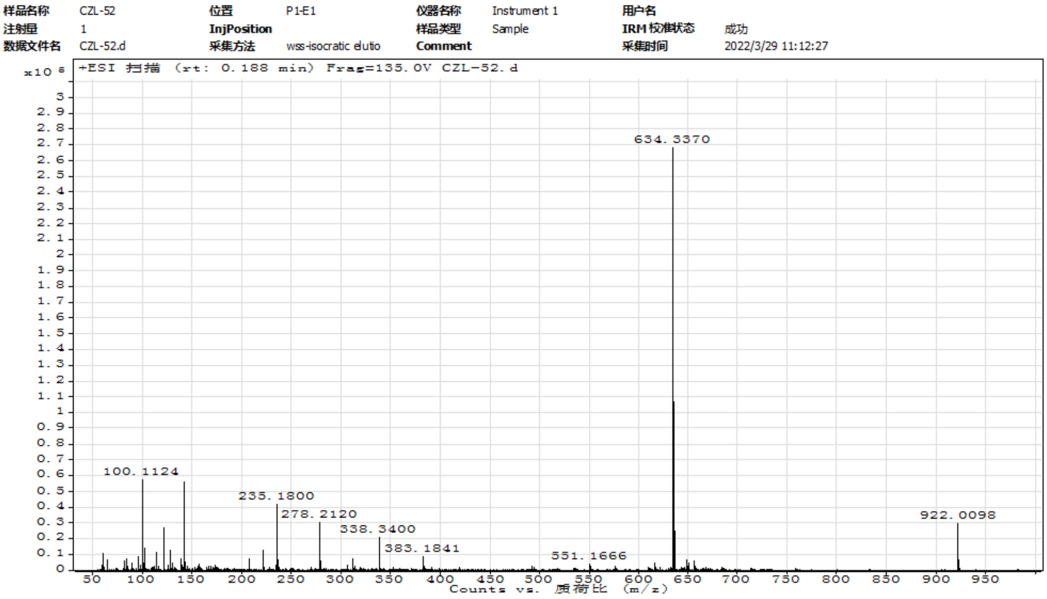

Figure S15. The ^{1}H-NMR spectrum of compound **3** in $CDCl_3$

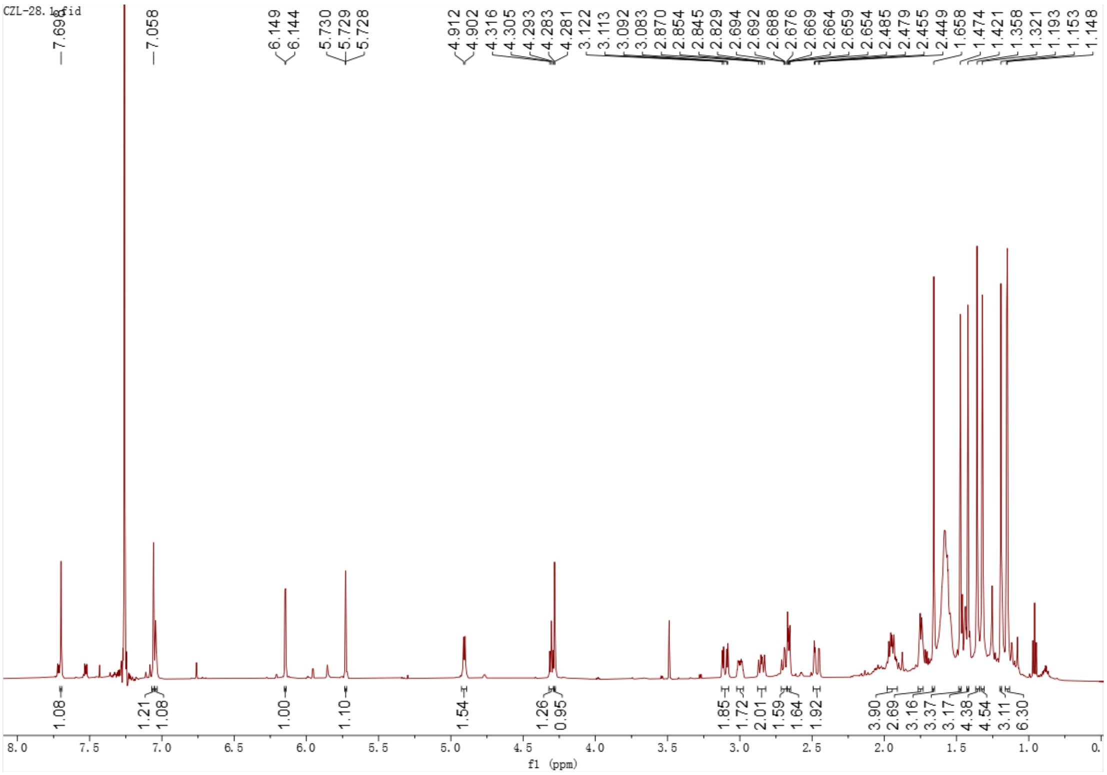

Figure S16. The ^{13}C-NMR spectrum of compound **3** in $CDCl_3$

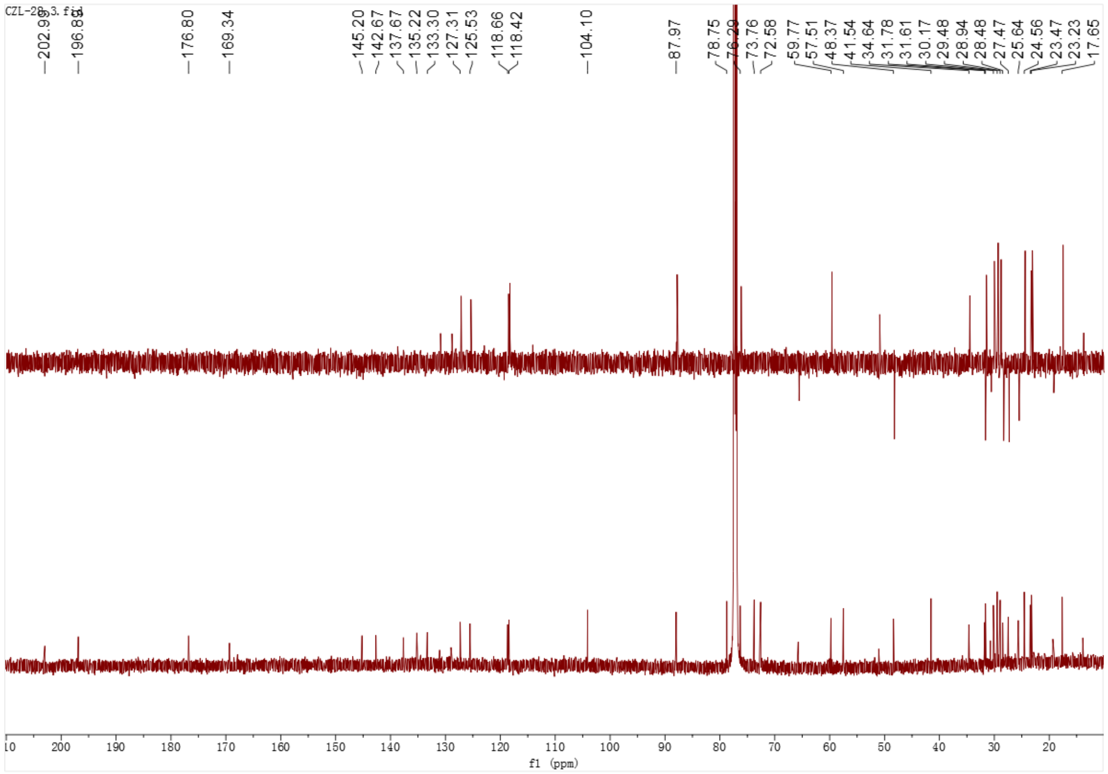

Figure S17. The HSQC spectrum of compound **3** in $CDCl_3$

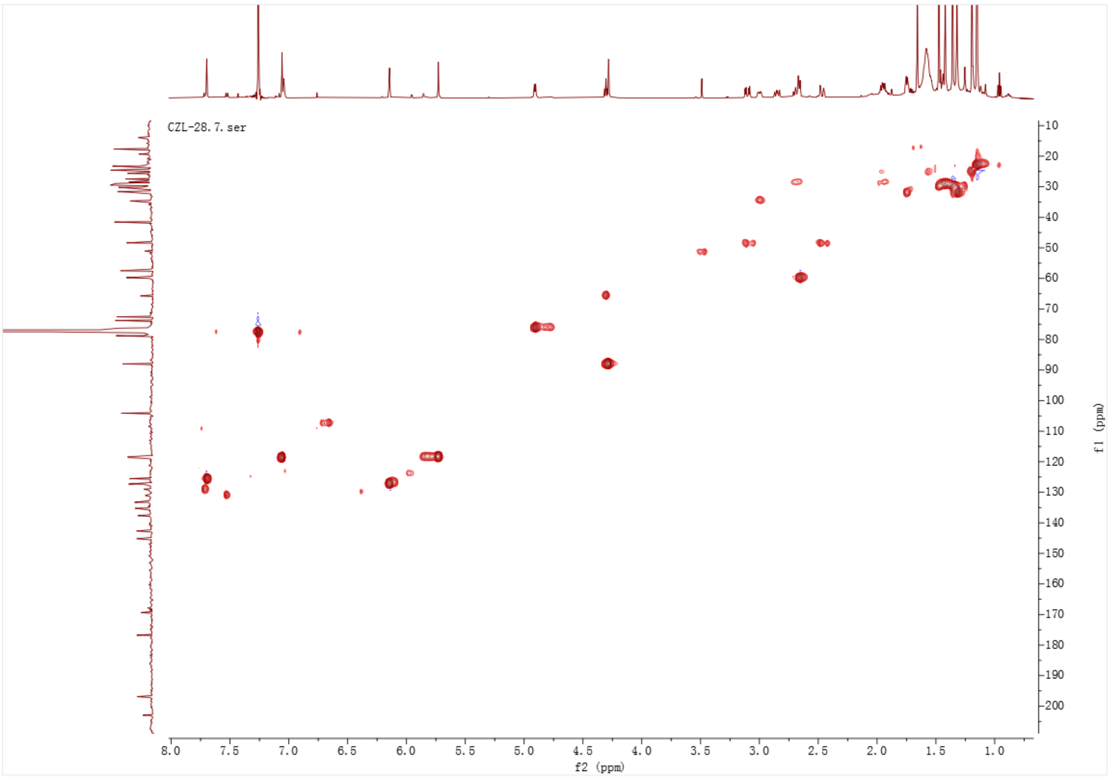

Figure S18. The ^1H-^1H COSY spectrum of compound **3** in $CDCl_3$

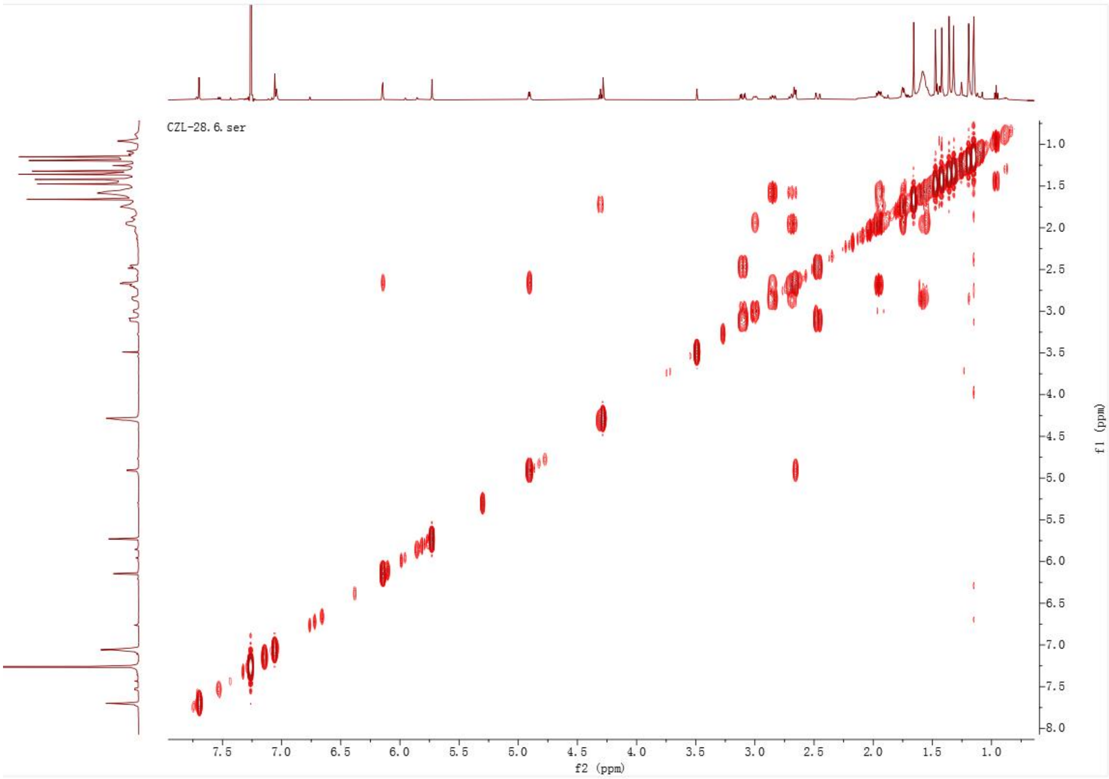

Figure S19. The HMBC spectrum of compound **3** in $CDCl_3$

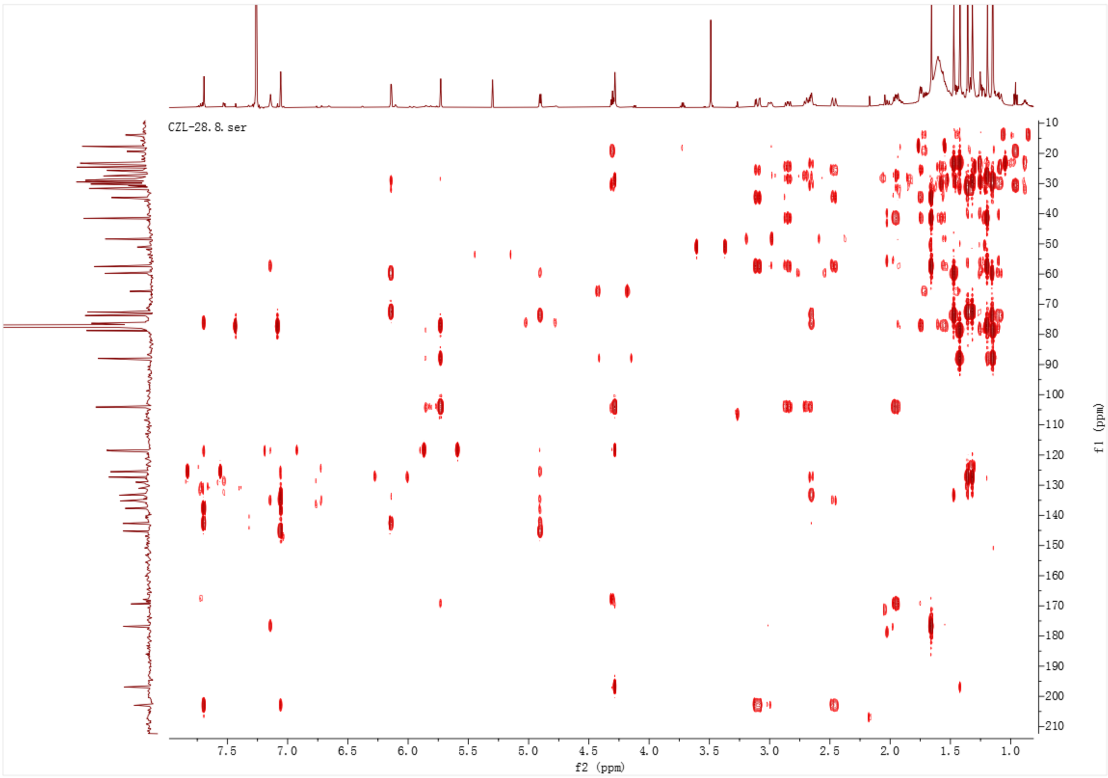

Figure S20. The NOESY spectrum of compound **3** in $CDCl_3$

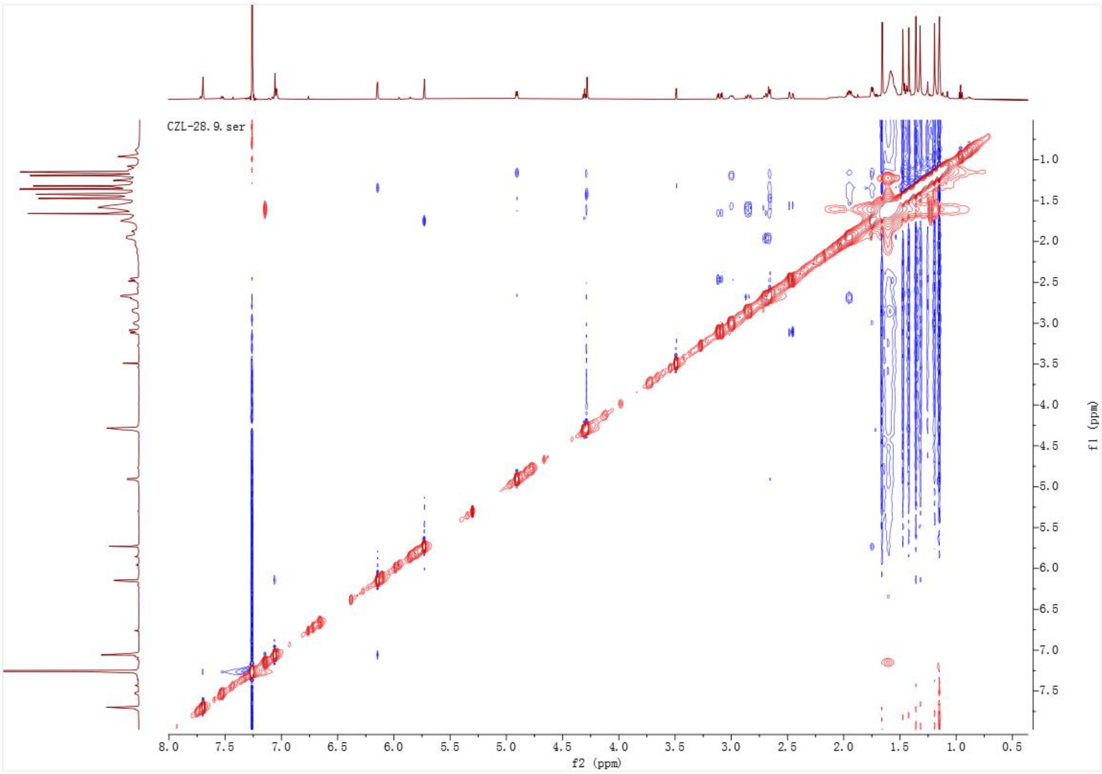

Figure S21. The (+)-HR-ESIMS spectrum of compound **3**

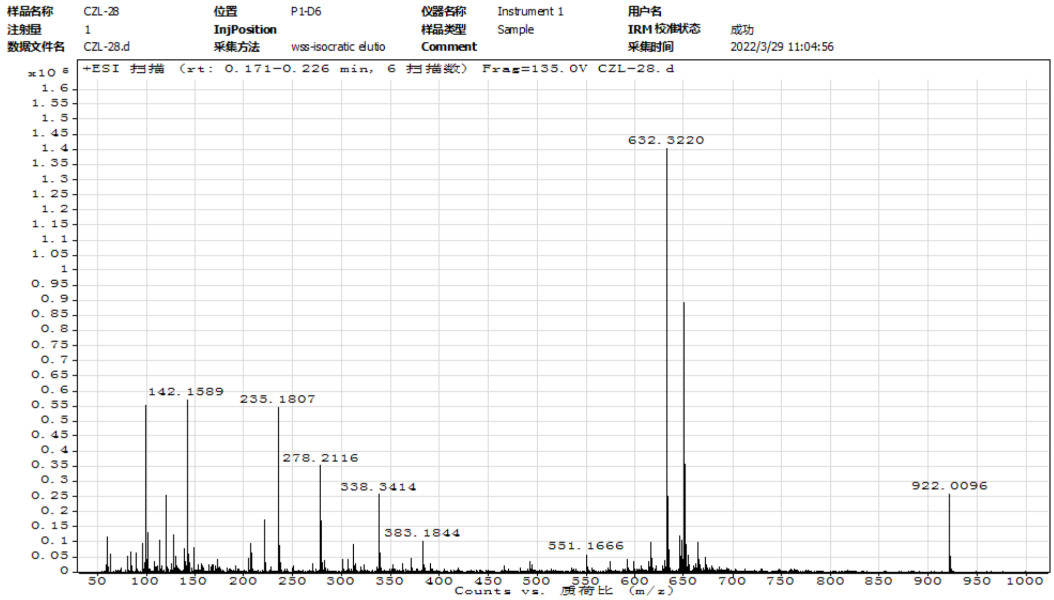

Figure S22. The ^{1}H-NMR spectrum of compound **4** in $CDCl_3$

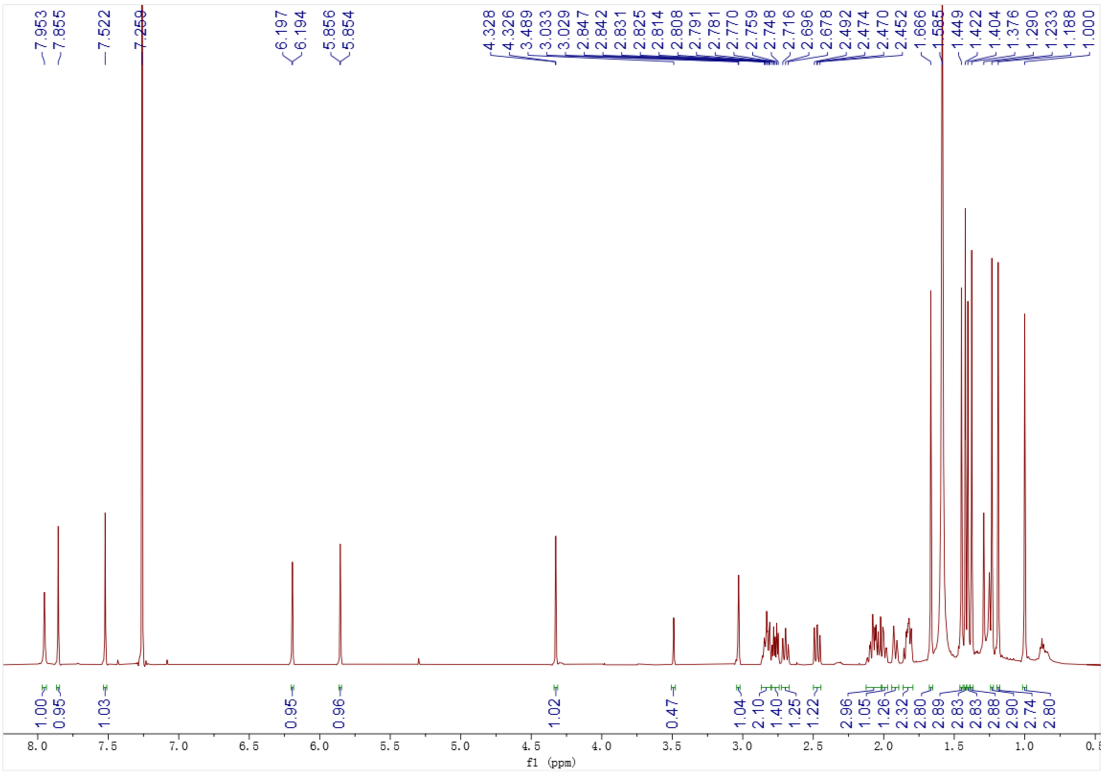

Figure S23. The ^{13}C-NMR spectrum of compound **4** in $CDCl_3$

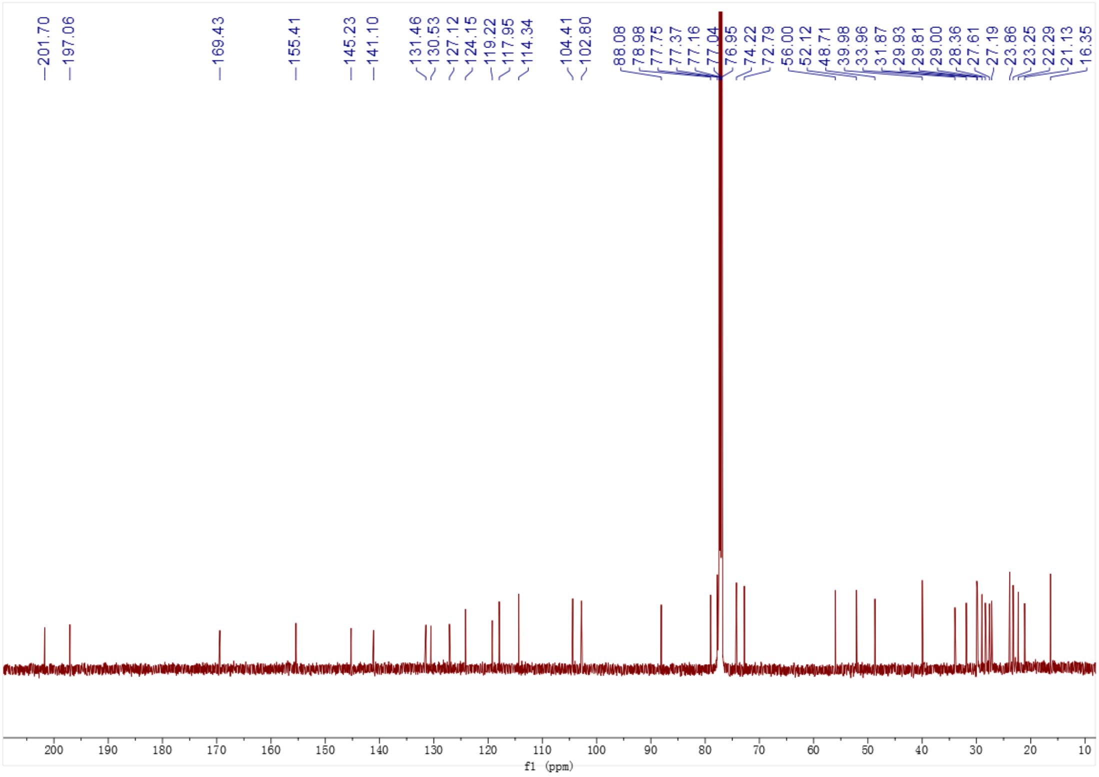

Figure S24. The HSQC spectrum of compound **4** in $CDCl_3$

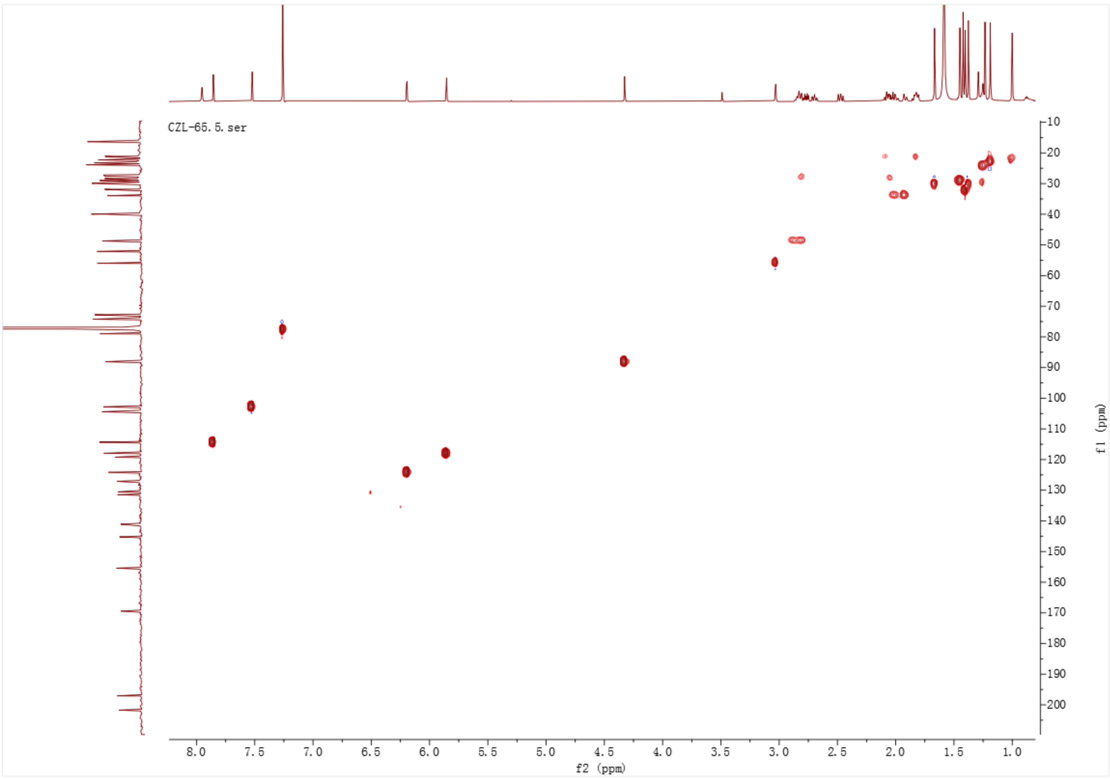

Figure S25. The ^{1}H-^{1}H COSY spectrum of compound **4** in $CDCl_3$

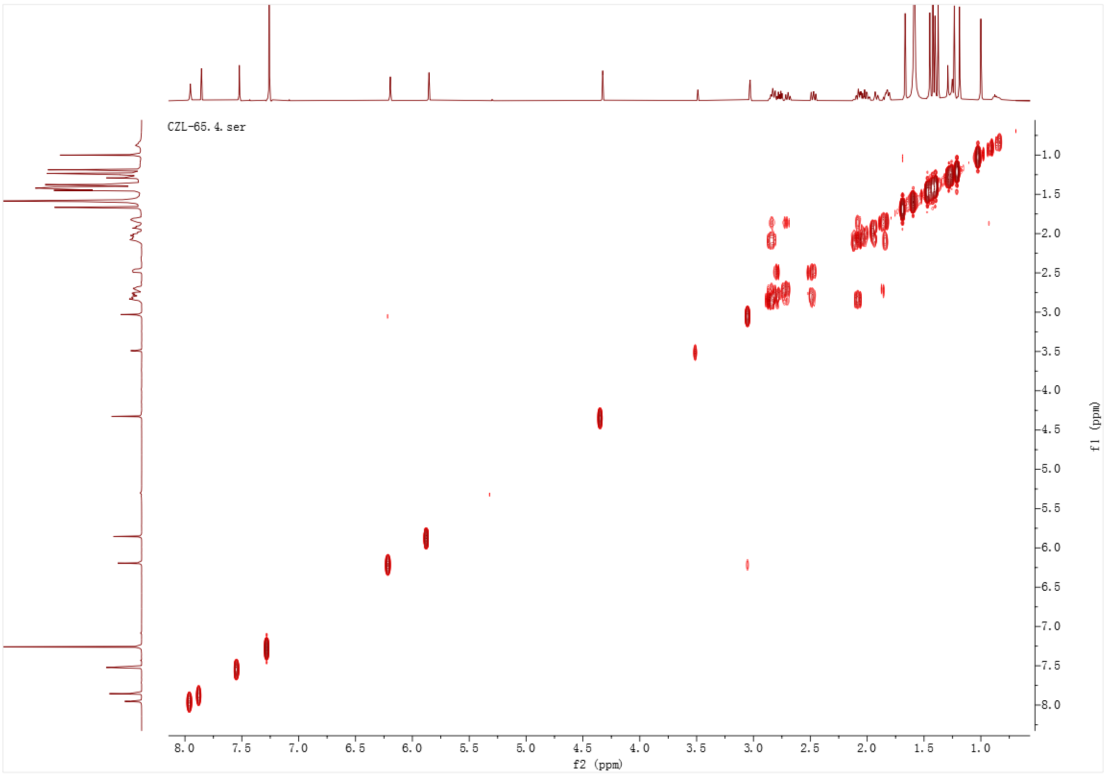

Figure S26. The HMBC spectrum of compound **4** in $CDCl_3$

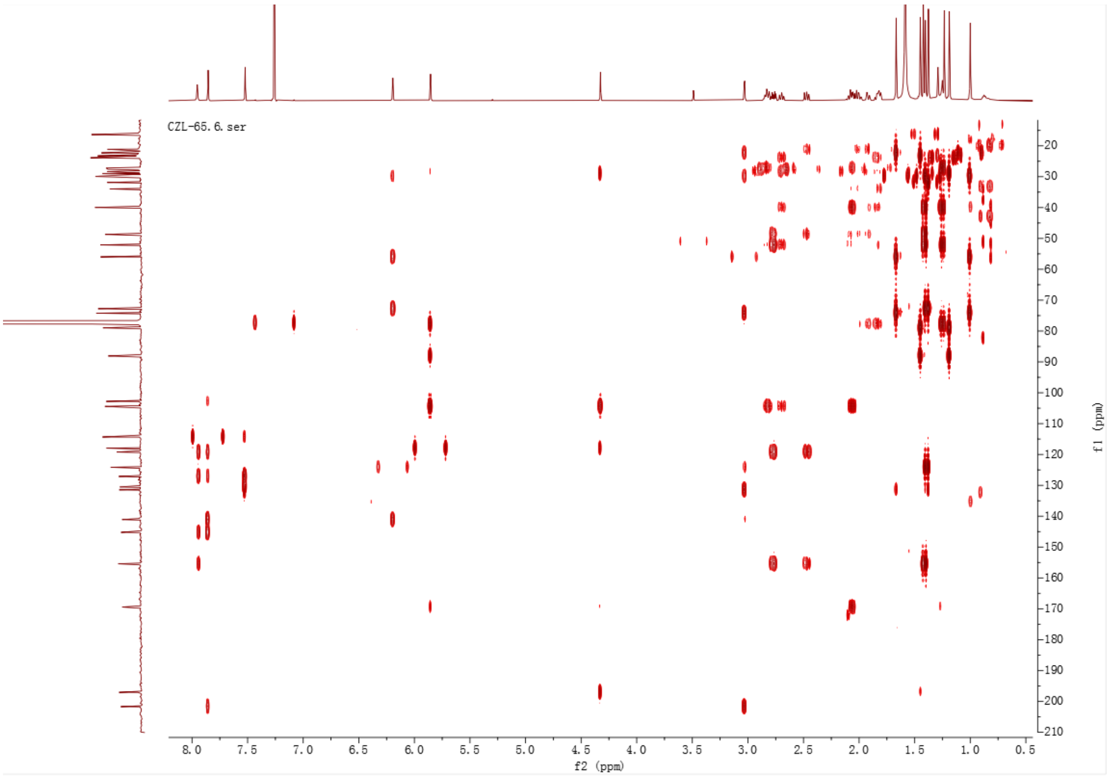

Figure S27. The NOESY spectrum of compound **4** in $CDCl_3$

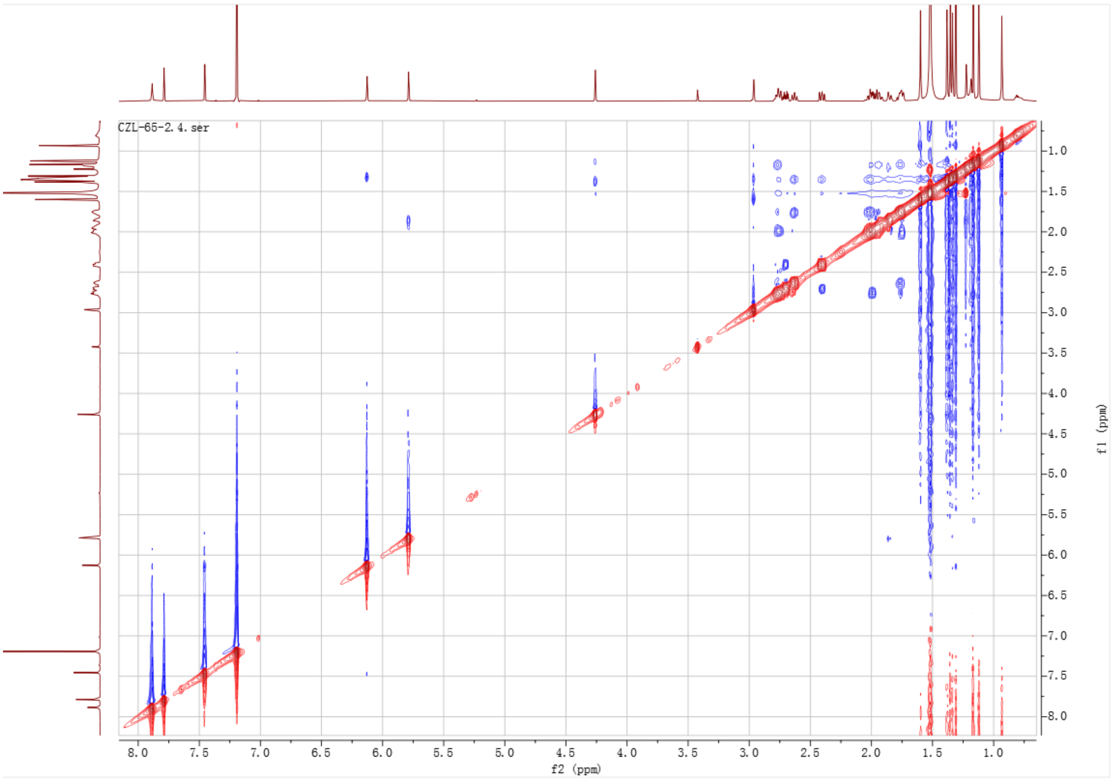

Figure S28. The (+)-HR-ESIMS spectrum of compound **4**

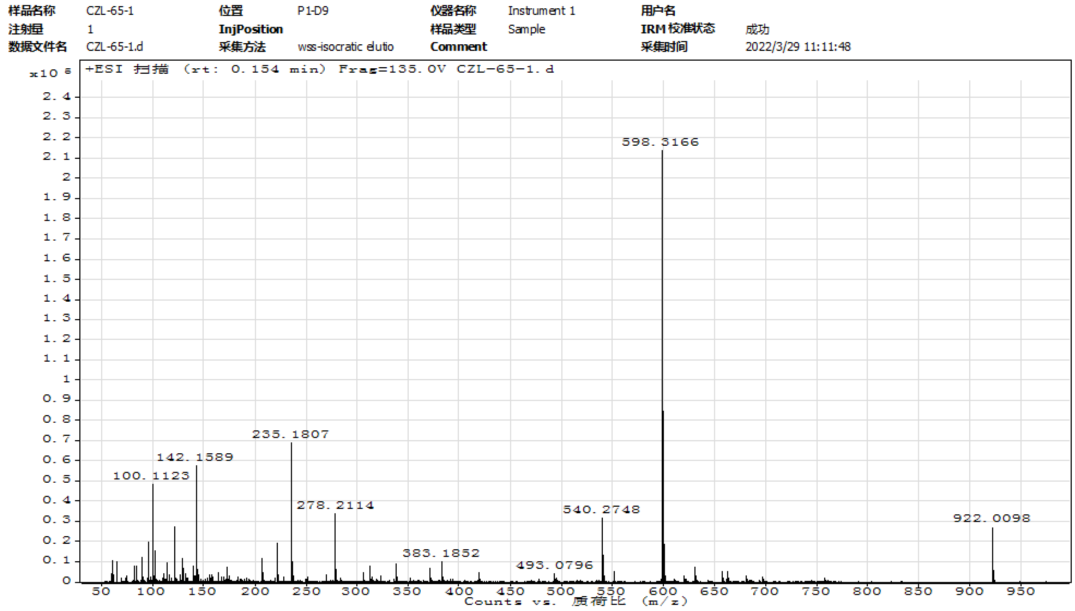

Supplement: Supplementary file 1 [file marinedrugs-21-00593-s001.zip › marinedrugs-2687748-supplementary.pdf]
